# Nonstructural N- and C-tails of Dbp2 confer the protein full helicase activities

**DOI:** 10.1016/j.jbc.2023.104592

**Published:** 2023-03-08

**Authors:** Qin-Xia Song, Na-Nv Liu, Zhao-Xia Liu, Ying-Zi Zhang, Stephane Rety, Xi-Miao Hou, Xu-Guang Xi

**Affiliations:** 1 College of Life Sciences, State Key Laboratory of Crop Stress Biology in Arid Areas, Northwest A&F University, Yangling, Shaanxi, PR China; 2 University. Lyon, ENS de Lyon, University. Claude Bernard, CNRS UMR 5239, INSERM U1210, LBMC, Lyon, France; 3 Laboratoire de Biologie et Pharmacologie Appliquée (LBPA), UMR8113 CNRS, ENS Paris-Saclay, Université Paris-Saclay, Gif-sur-Yvette, France

**Keywords:** RNA helicase Dbp2, disordered protein regions, RNA-protein interaction, X-ray crystallography, FRET

## Abstract

Human DDX5 and its yeast ortholog Dbp2 are ATP-dependent RNA helicases that play a key role in normal cell processes, cancer development, and viral infection. The crystal structure of the RecA1-like domain of DDX5 is available but the global structure of DDX5/Dbp2 subfamily proteins remains to be elucidated. Here, we report the first X-ray crystal structures of the Dbp2 helicase core alone and in complex with ADP at 3.22 Å and 3.05 Å resolutions, respectively. The structures of the ADP-bound post-hydrolysis state and apo-state demonstrate the conformational changes that occur when the nucleotides are released. Our results showed that the helicase core of Dbp2 shifted between open and closed conformation in solution but the unwinding activity was hindered when the helicase core was restricted to a single conformation. A small-angle X-ray scattering experiment showed that the disordered amino (N) tail and carboxy (C) tails are flexible in solution. Truncation mutations confirmed that the terminal tails were critical for the nucleic acid binding, ATPase, and unwinding activities, with the C-tail being exclusively responsible for the annealing activity. Furthermore, we labeled the terminal tails to observe the conformational changes between the disordered tails and the helicase core upon binding nucleic acid substrates. Specifically, we found that the nonstructural terminal tails bind to RNA substrates and tether them to the helicase core domain, thereby conferring full helicase activities to the Dbp2 protein. This distinct structural characteristic provides new insight into the mechanism of DEAD-box RNA helicases.

DEAD-box RNA helicases represent the largest helicase family in the helicase superfamily 2 (1). The DEAD-box family is implicated in many aspects of RNA metabolism, such as remodeling the RNA–protein complex, ribosome biogenesis, mRNA and microRNA processing, RNA editing, and RNA degradation (2). Human DDX5, yeast Dbp2, and fly Rm62 represent a subfamily of DEAD-box proteins. Dbp2 is a *bona fide* RNA helicase that is now known to be involved in multiple pathways of RNA metabolism. It has been implicated in cotranscriptional mRNA-binding protein assembly (3) and facilitates efficient transcription termination *via* the Nrd1–Nab3–Sen1 complex (4). In particular, Dbp2 regulates lncRNA-DNA hybrid and R-loop formation and loss of Dbp2 leads to the accumulation of RNA-DNA hybrids *in vivo* (5). Moreover, Dbp2 possesses RNA-DNA unwinding and annealing activities *in vitro* (6). Given that DDX5 resolves R-loops at DNA double-strand breaks to promote DNA repair (7), Dbp2 may be involved in DNA double-strand break repair. Dbp2 also can disrupt RNA and DNA G-quadruplex (G4) structures with two G-tetrad stacks (8); G4 sequences are widely present in various virus genomes (9) and Dbp2 is also implicated in RNA virus infections (10). Moreover, the abnormal expression of DDX5 appears in various cancer cells, such as breast cancers (11), colon cancers (12), prostate cancer (13), lung cancer (14), gastric cancer (15), and leukemia (16). Therefore, DDX5 is a promising drug target for cancer diagnosis. In cells lacking Dbp2, ectopic expression of DDX5 could rescue defects in growth and gene expression (17). The complementation of Dbp2 with DDX5 illustrates the functional conservation of DDX5 and Dbp2 across evolution and confirms that the DDX5/Dbp2 subfamily shares highly conserved protein sequences and cellular functions.

Similar to other DEAD-box proteins, DDX5/Dbp2 is composed of two RecA-like domains that include 11 conserved motifs. Motifs Q, I, Ia, GG, Ib, II, and III are found in the first RecA-like domain (also named the DEAD domain or RecA1) and motifs IV, V, QxxR, and IV are found in the second RecA-like domain (also named cHelicase or RecA2), these motifs are involved in RNA and ATP binding (18). In addition to the conserved helicase core, the N- and C-terminal tails of DEAD-box proteins are highly variable, giving them a role in augmenting enzymatic activity or regulating specific protein and nucleic acid interactions (19, 20). N- and C-tails of DEAD-box proteins commonly contain low-complexity sequences (21). Low-complexity sequences rich in arginine and glycine, called the RGG motif regulate nucleic acid and protein interactions through arginine methylation and partner-binding proteins (22). Dbp2 contains two RGG motifs located in the N- and C-tails, including the C-terminal RGG motif which is critical for binding and destabilizing G4 DNA and RNA substrates (23). Dbp2 harbors two inverse activities, ATP-dependent unwinding and ATP-independent annealing activities but which domains perform these functions is still unclear. In particular, more detail is needed on the role of the N- and C-tails of Dbp2 in RNA remodeling.

Understanding the tertiary structure of Dbp2 is critical in the search for small molecule inhibitors of DDX5-associated cancers. Currently, only the crystal structure of the RecA1 domain (residues 79–303) of DDX5 is available. Here, we report the crystal structure of the Dbp2 helicase core, which consists of two RecA-like domains and N-/C-terminal extensions (NTE/CTE). A cyclic open-closed motion of the Dbp2 helicase core is required for rapid and efficient unwinding activity. Our structural-guided truncated mutants in combination with nucleic acid binding, ATPase activity, unwinding, and annealing studies show that the helicase core itself has no binding, unwinding, and annealing activities. In contrast, the terminal tails are essential for nucleic acid binding and ATPase activity and regulate the unwinding; the C-tail is specifically responsible for annealing. Furthermore, the intrinsically disordered N- and C-tails become compact upon RNA binding and RNA substrates induce conformational changes that confer full helicase activities to the Dbp2 protein. This systematic and thorough study revealed unique structural characteristics of Dbp2, thus providing invaluable insights into the mechanism of the DEAD-box protein.

## Results

### Dbp2 is an RNA unwinding helicase, particularly efficient on the forked RNA/DNA duplex unwinding

**Figure 1:**
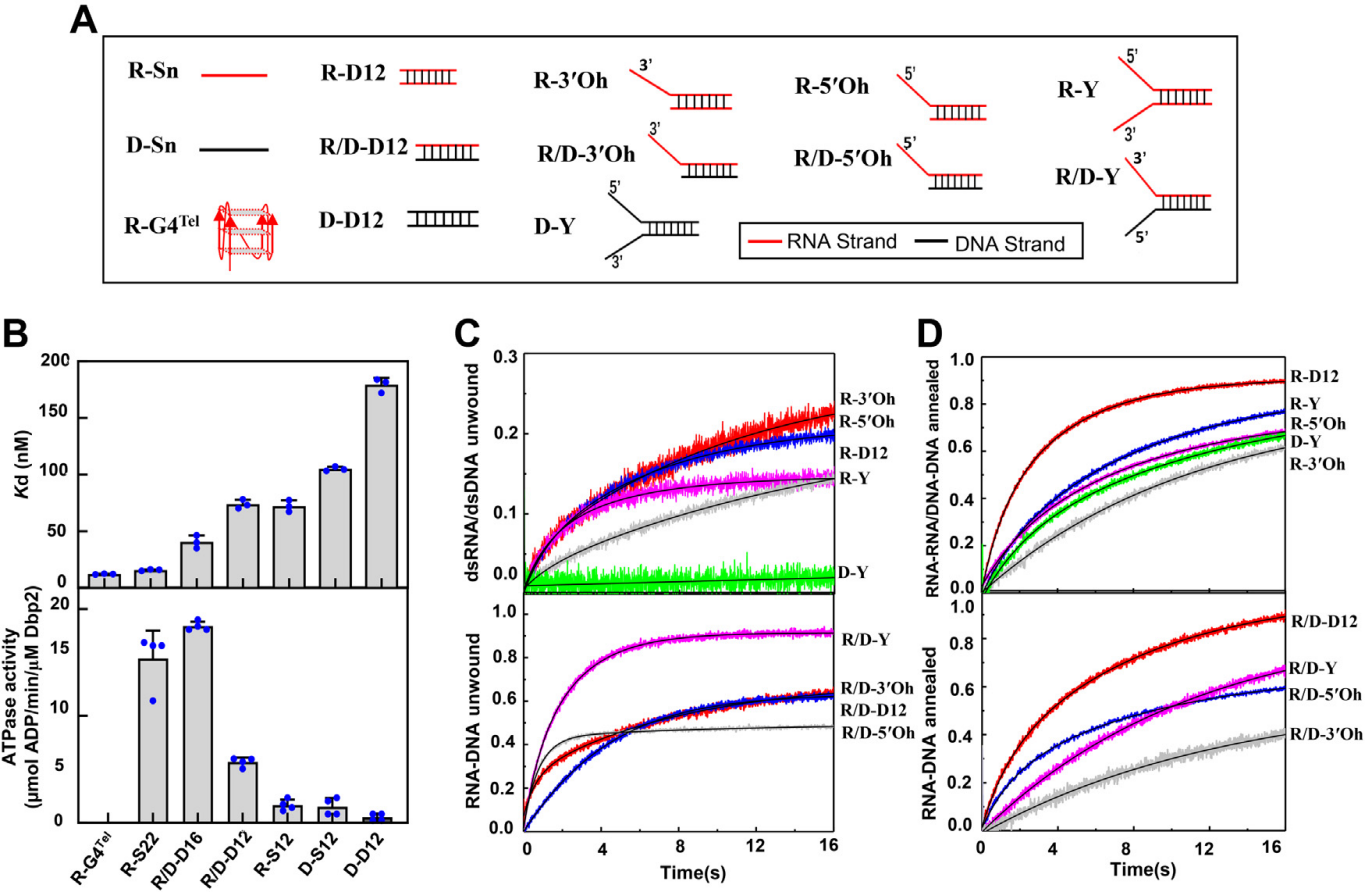
**Biochemical characterization of Dbp2.***A*, schematic presentation of the RNA and DNA substrates used in the binding, ATPase, unwinding, and annealing experiments. S represents a single strand; n, the length of single and duplex strands; -D, duplex strand; Oh, overhanging strand; Y, fork-structured strand. *B*, upper panel, the binding affinities of different substrates to Dbp2. The *K*_d_ values were measured in three independent replicates; bottom panel, ATPase activities of Dbp2 in the presence of different nucleic acids, error bars represent SD from four independent reactions. *C*, Dbp2-catalyzed unwinding kinetics with RNA and DNA duplex substrates and RNA/DNA hybrid duplexes. *D*, Dbp2-catalyzed annealing kinetics with RNA-RNA strands and RNA-DNA strands. A stopped-flow kinetic trace was fitted from the average of 10 individual traces, data show mean ± SD from three independent experiments.

Prior to characterizing the structure of the Dbp2 protein, we first investigated the intrinsic enzymatic activities of a highly purified recombinant Dbp2 from *Saccharomyces cerevisiae*. We used a fluorescence anisotropy assay with various RNA, DNA, and hybridized RNA/DNA (R/D) substrates to gain insight into the binding properties of Dbp2 (Fig. 1*A*). Apparent dissociation constants (*K*_d_) from the protein concentration-dependent changes in fluorescence anisotropy show that Dbp2 displayed significantly higher binding affinities to the 22 nt of ssRNA (R-S22) and RNA G4 (R-G4^Tel^) than to short length ssRNA (R-S12) and ssDNA (D-S12). Dbp2 showed relatively weak binding to dsDNA (D-D12) (Fig. 1*B*, upper panel; Table S1).

The RNA-dependent ATPase activities of Dbp2 were measured using a coupled spectrophotometric ATPase assay. In the absence of RNA, Dbp2 had undetectable ATPase activity that was stimulated by the addition of R-S22 (15.60 ± 2.34 μmol ADP/min/μM Dbp2) or a 16 bp RNA/DNA duplex (R/D-D16; 18.39 ± 0.42 μmol ADP/min/μM Dbp2) (Fig. 1*B*, bottom panel; Table S1). Other short DNA or RNA substrates (R/D-D12, R-S12, D-S12, and D-D12) resulted in very weak ATPase activity (1.04 ± 0.51∼5.68 ± 0.43 μmol ADP/min/μM Dbp2) in agreement with the binding studies.

The helicase activity of Dbp2 was further characterized with respect to substrate specificity and directionality. A stopped-flow fluorescence assay was used to determine the unwinding amplitudes (Am) and rates of different RNA duplexes and R/D hybrid duplexes. As shown in Figure 1*C*, the results indicate that Dbp2 generally displayed higher unwinding amplitudes and rates toward R/D duplexes than to RNA duplexes. Although the forked DNA duplex (D-Y) was not an appropriate substrate, unwinding of RNA duplexes occurred with both 3′ and 5′ overhang substrates, indicating that Dbp2 does not have polarity preference (comparison of substrate R-3′Oh *versus* R-5′Oh). Interestingly, Dbp2 displayed its highest unwinding amplitude and rates on the forked R/D duplex (R/D-Y). Altogether, these results indicate that Dbp2 is an RNA unwinding helicase, particularly the forked RNA/DNA duplex.

Furthermore, Dbp2 facilitates not only the disruption but also the formation of RNA duplexes (6). Using a stopped-flow FRET experiment, we tested the annealing kinetics of Dbp2 for different types of substrates (Fig. 1*D* and Table S1). The results show that Dbp2 more efficiently annealed blunt-end RNA-RNA and RNA-DNA substrates than other types of substrates. These results together demonstrate that Dbp2 is an active RNA helicase *in vitro*.

### Crystal structure of Dbp2 catalytic core

To obtain structural information on the DDX5/Dbp2 subfamily, Dbp2 from *S. cerevisiae* was used for a crystallographic study. Dbp2 shares 45.7% sequence identity with human DDX5 (Fig. S1). Full-length Dbp2 and several different truncated constructions were expressed and purified for crystallization but no crystals were obtained. Therefore, the full-length Dbp2 underwent limited proteolysis and a compact segment was purified for the crystallographic study (Fig. S2).

**Figure 2:**
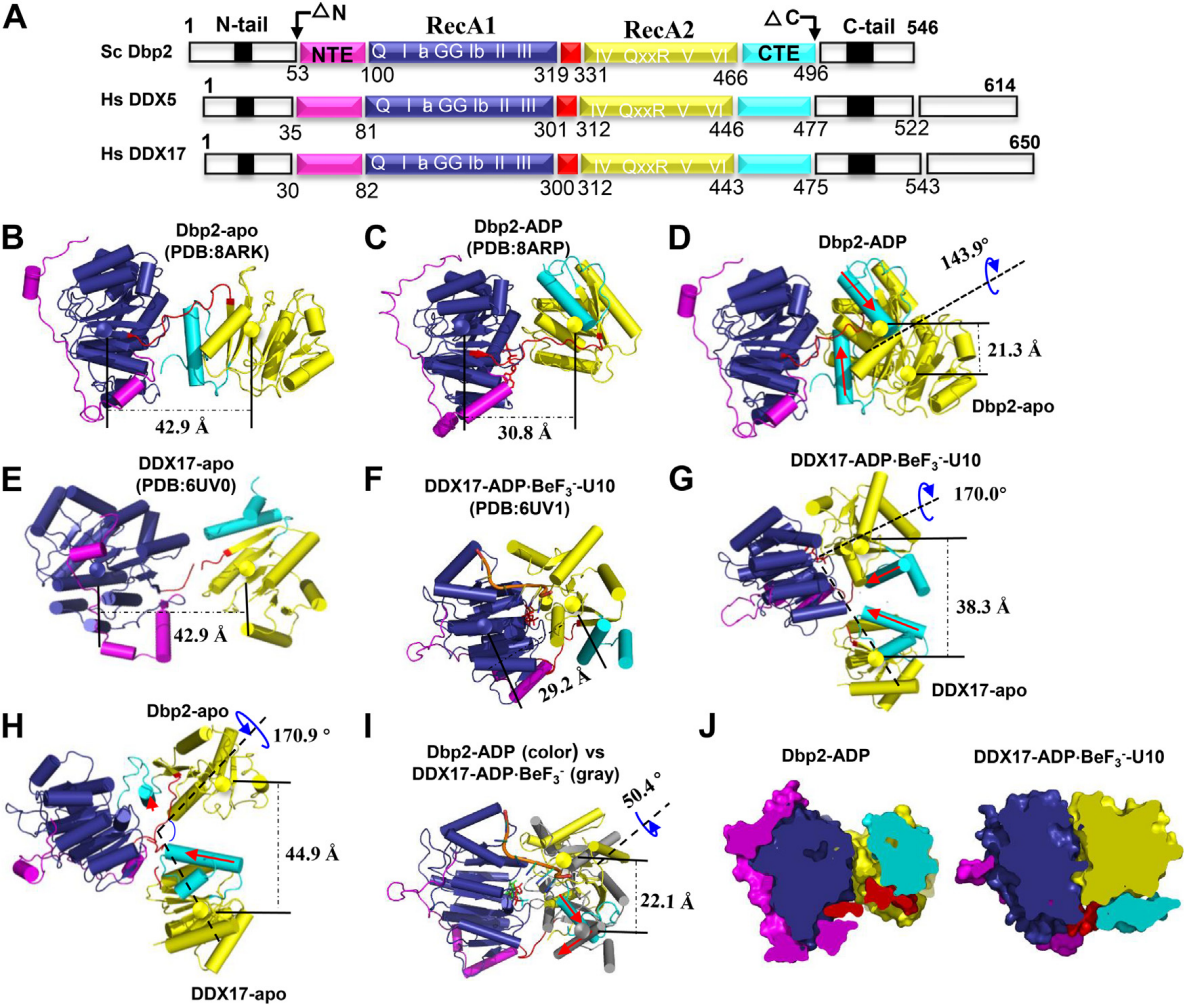
**Overall structure of the Dbp2 catalytic core apo and ADP-bound state in *Saccharomyces cerevisiae***. *A*, a schematic of the domains of Dbp2/DDX5/DDX17 proteins. Arrows show the truncated protein used for crystallization. The amino- (N-) tail (aa 1–52) and carboxy- (C-) tail (aa 497–546) are shown in *white*, NTE (aa 53–100) in *magenta*, RecA1 (aa 101–319) in *deep blue*, linker (aa 320–331) in *red*, RecA2 (aa 332–466) in *yellow*, CTE (aa 467–496) in *cyan* (these same colors are used in other figures). *B* and *C*, the crystal structures of Dbp2-apo and ADP-bound state after superposition of the RecA1 domains. The structures are shown as cartoon models; the center of mass of the RecA-like domain is shown in the sphere model; the *dotted line* shows the distance between RecA1 and RecA2. *D*, conformational comparison of Dbp2-apo and Dbp2-ADP after superposition of the RecA1 domains. The *blue arrow* shows the rotation angle of RecA2 domain in both structures; the *dotted line* shows the distance of RecA2 in both structures. *E* and *F*, crystal structures of DDX17-apo and DDX17/ADP·BeF_3_^-^ after superposition the RecA1 domains. *G*, conformational comparison of DDX17-apo and DDX17-ADP·BeF_3_^-^-U10 after superposition of the RecA1 domains. *H*, conformational comparison of Dbp2-apo and DDX17-apo after superposition of the RecA1 domains. *I*, conformational comparison of Dbp2-ADP and DDX17-ADP·BeF_3_^-^ after superposition of the RecA1 domains. *J*, cut-away view of Dbp2-ADP and DDX17-ADP·BeF_3_^-^-U10. CTE, C-terminal extension; NTE, N-terminal extension.

The crystal structures of Dbp2 catalytic core apo and ADP-bound states were solved by molecular replacement and refined to 3.22 Å and 3.05 Å resolutions, respectively (Table S2), using the N-terminal domain of DDX5 as a search model (PDB 4A4D). The helicase core of Dbp2 consists of two RecA-like domains (RecA1: aa 101–319; RecA2: aa 332–465), an NTE (aa 53–100), a CTE (aa 466–496), and a linker (aa 320–331) (Fig. 2*A*). The Dbp2 catalytic core apo (Dbp2-apo) crystallized in space group *C*121 with 2.5 molecules in the asymmetric unit: two molecules (aa 57–496) in nearly identical conformation (rmsd 1.11 Å) and a half molecule (aa 57–326). This molecule fragment composed of NTE-RecA1 is likely generated by the limited proteolysis of the linker between the RecA1 and RecA2 domains. The Dbp2 catalytic core bound with ADP (Dbp2-ADP) crystallized in space group *P*2_1_2_1_2_1_ with six molecules (aa 53–496) in the asymmetric unit, arranged in a D3 hexamer (Fig S4*A*). For Dbp2-ADP structure, because of the crystals suffered from severe twinning and the structure was difficult to refine, we collected many crystals prepared in different conditions. Finally, we obtained the crystal of Dbp2 in complex with ADP and a 16 nt polyGT ssDNA (ssDNA is not visible in the density). ADP is no clearly visible after molecular replacement in Fo-Fc difference map but could be refined with good density (Final 2Fo-Fc map). Density for ADP was verified with calculation of a composite omit map (Fig. S3), meanwhile, comparison of ADP site with DDX17 structure (Protein Data Bank 6uv3) showed a good conservation of the nucleotide-binding site (Fig S4*B*).

In the crystal structure of Dbp2-apo, two RecA-like domains adopted an open state, with the NTE subdomain located on the exterior surface of RecA1 and the CTE subdomain inserted between two RecA-like domains (Fig. 2*B*). To estimate the position and intrinsic mobility of the two RecA-like domains, we calculated the distance between the centers of mass of the RecA1 and RecA2. This distance was 42.9 Å in the open state, showing that the two domains were far apart, as shown in Figure 2*B*. A flexible linker connects to the core domain but the conformation of Dbp2-apo is rigid. In the Dbp2-apo structure, the RecA1 and RecA2 domains are restrained by the linker and CTE subdomains. The RecA1, RecA2, NTE, CTE subdomains, and linker form an interaction network, stabilizing the conformation (Fig S4*C*).

When Dbp2 bound to ADP, the two RecA-like domains underwent a large conformational change, distance between the centers of mass of the RecA1-RecA2 domains was reduced to 30.8 Å (Fig. 2*C*) and the RecA2 domain rotated by 143.9° (Fig. 2*D*). Thus, in presence of ADP, the RecA2 domain moved closer to the RecA1 domain and the helicase core adopted a more compact conformation than in the Dbp2-apo structure. Small-angle X-ray scattering (SAXS) analysis showed that Dbp2-ADP is monomeric in solution (Table S3) and the observed hexamer form is probably due to crystal packing. Although the conformation of the helicase core in the crystal structure of Dbp2-ADP was in a semi-closed state, it was in a completely open state in solution (Fig S4, *D* and *E*). Previous studies have shown that conformation of the helicase core exists in the preferred orientation and moves independently (24). Hence, both conformations may exist upon the binding of ADP.

Based on the structural homology alignment with DALI (http://ekhidna.biocenter.helsinki.fi/dali_server/), the closest structure to Dbp2 is the DEAD-box protein DDX17 (25), the human paralog of DDX5, which has similar roles in RNA metabolism and cancer progression (26). As expected, the intrinsic mobility of the RecA2 domain was observed in the unbound and ATP-bound states of DDX17, and the distance between the centers of mass of the RecA1-RecA2 domains shifted from 42.9 Å to 29.2 Å (Fig. 2, *E* and *F*), while the RecA2 domain rotated by 170.0°. Moreover, the conformational change of RecA2 between Dbp2-apo and Dbp2-ADP was also similar to the difference between DDX17-apo and DDX17-ADP·BeF_3_^-^-U10 (Fig. 2, *D* and *G*). Then, a comparison of the position of the RecA2 domain in the Dbp2-apo and DDX17-apo structures showed a difference in the distance between the center of mass of RecA2 of 44.9 Å and a difference in orientation of 170.9° (Fig. 2*H*). Although the apo-structures of Dbp2 and DDX17 were both in the open state, the conformation of the helicase core was different in the RecA2 position. In contrast, comparing structures of Dbp2-ADP and DDX17-ATP analog (Fig. 2*I*), the distance between the center of mass of RecA2 of both structures was only 22.1 Å and the angle between the RecA2 domains was 50.4°, showing that both conformations are closer than conformations between Dbp2-apo and DDX17-apo structures. Furthermore, we found that the P loop of these structures is associated with adenosine nucleotides. Thus, we compared the P loop of Dbp2 and DDX17 with or without ADP and ADP·BeF_3_^-^, demonstrating that the P loop gradually moves to the left in the presence of the adenosine nucleotide and with an increase in the number of phosphate groups (Fig S4*F*). These structures show that the helicase core of Dbp2/DDX17 alone tends to be in a fully open state, but, in the presence of adenosine nucleotides, the helicase core tends to be in a closed state.

### The NTE and CTE of Dbp2 regulate ATPase activity

**Figure 3:**
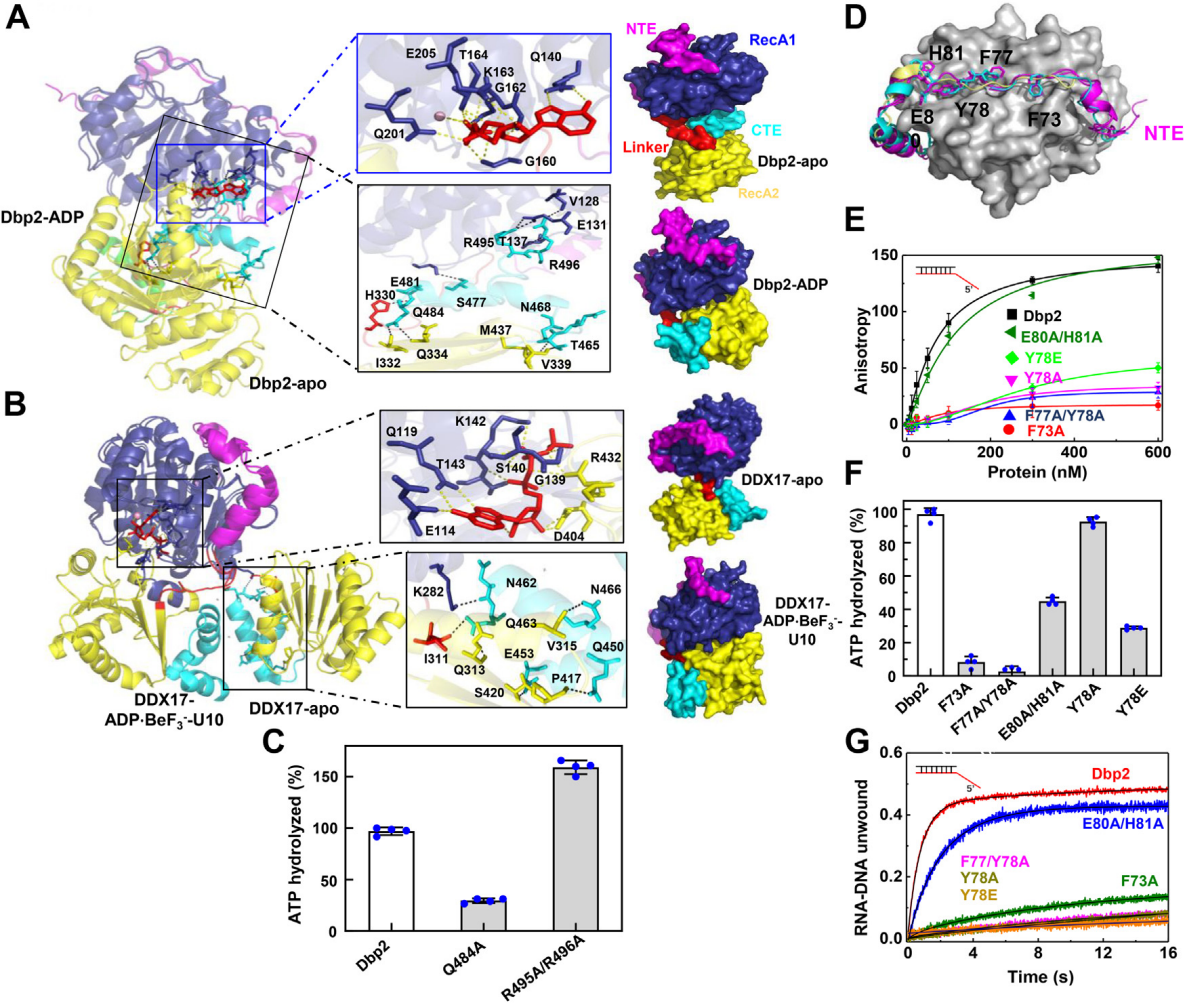
**The N- and C-terminal extensions of Dbp2 regulate ATPase activity.***A*, the CTE wedges between the helicase domain occupying the location of the ADP site in the Dbp2-apo structure and the ADP-binding sites in the Dbp2-ADP structure. The residues involved in the interaction are shown in the middle box. The right panel represents the overall structure of Dbp2-apo and Dbp2-ADP as molecular surfaces. ADP is shown as a *red stick*. *B*, the positions of CTE in the DDX17-apo structure and the ADP·BeF_3_^-^-binding sites in the DDX17-ADP·BeF_3_^-^-U10 structure. Residues involved in the interaction are shown in the middle box. The right panel represents the overall structures of DDX17-apo and DDX17-ADP·BeF_3_^-^-U10 as molecular surface. ADP·BeF_3_^-^ is shown as a *red stick*. *C*, ATPase activity of WT Dbp2 and mutant full-length Dbp2. *D*, overlay of NTE of Dbp2-apo and Dbp2-ADP (*magenta*) with the same region in DDX17 (*cyan*) and DDX5 (*pale yellow*). The conserved amino acids are shown as a stick model. *E*–*G*, the binding affinities (*E*) and ATPase (*F*) and unwinding (*G*) activities of WT Dbp2 and mutant full-length Dbp2. Data show mean ± SD from four (*C* and *F*) and three (*E* and *G*) independent experiments. CTE, C-terminal extension; NTE, N-terminal extension.

The overall structure of the Dbp2 helicase core is similar to that of other DEAD-box proteins and in particular to the structure of DDX17. However, the relative orientation and location of the NTE and CTE showed differences in detail. In the structure of Dbp2-apo, the CTE consists of an α-helix and a disordered loop that inserts into the cleft of the helicase core. Residues R495 and R496 of the CTE form five hydrogen bonds with V128, E131, and T137 of the Q motif in the RecA1 domain (Fig. 3*A*, black box). Simultaneously, we observed the interaction of ADP with the Q motif of the RecA1 domain in the Dbp2-ADP structure (Fig. 3*A*, blue box). Structural superposition showed that residues R495 and R496 occupy the ADP binding site, and ADP binding resulted in the CTE moving to the side of the RecA2 domain (Figs. 2*J* and 3*A*). Further, the R495A/R496A double mutant also showed increased ATPase activity *in vitro* (Fig. 3*C*). In contrast, the CTEs of both DDX17-apo and ATP-bound state were always on the side of the RecA2 domain (Fig. 3*B*). Moreover, we found multiple interactions in the CTE of the four structures, particularly with residue Q484 in Dbp2 corresponding to residue Q450 in DDX17, which is involved in conserved interactions in the four structures (Table S4). Mutant Q484A showed reduced ATPase activity compared with full-length Dbp2 (Fig. 3*C*). Therefore, our structural and *in vitro* biochemical studies both indicate that the CTE is critical in regulating the ATPase activity of Dbp2.

The NTE consists of universally conserved aromatic residues (Figs. 3*D* and S4*G*) and the orientation of the NTE found in the crystal structures of the Dbp2 catalytic core was similar to those in human DDX5 (27) and DDX17 (25). A previous study on DDX17 indicates that the NTE regulates ATPase activity (25); therefore, we tested whether the NTE of Dbp2 has a similar function. We investigated the roles of the conserved aromatic residues using a mutational analysis. The binding activities of mutants showed visibly reduced affinity, except for E80A/H81A (Fig. 3*E*). ATPase activity was abrogated for mutants F73A and F77A/Y78A, but activity in E80A/H81A showed a decrease of roughly 2.2-fold and in Y78E a decrease of 3-fold, but activity was nearly unaffected in Y78A (Fig. 3*F*). The unwinding activity was nearly abrogated in all mutants, except for E80A/H81A (Fig. 3*G* and Table S5). Interestingly, the Y56E mutant in DDX17 showed an increase in ATPase and unwinding activities, but the corresponding mutant Y78E in Dbp2 showed a severe decrease in ATPase activity and a complete loss of binding and unwinding activities. Because mutations can introduce disruptions in protein folding, we performed CD experiments to determine if the NTE and CTE mutants introduced changes in the secondary structure. The results showed that the CD spectra of mutants were similar to the WT Dbp2 (Fig S4, *H* and *I*). Taken together, our results indicate that the NTE is involved in the unwinding activity of Dbp2 by modulating both substrate binding and ATPase activity.

### Global conformational changes of RecA1 and RecA2 domain during RNA unwinding

**Figure 4:**
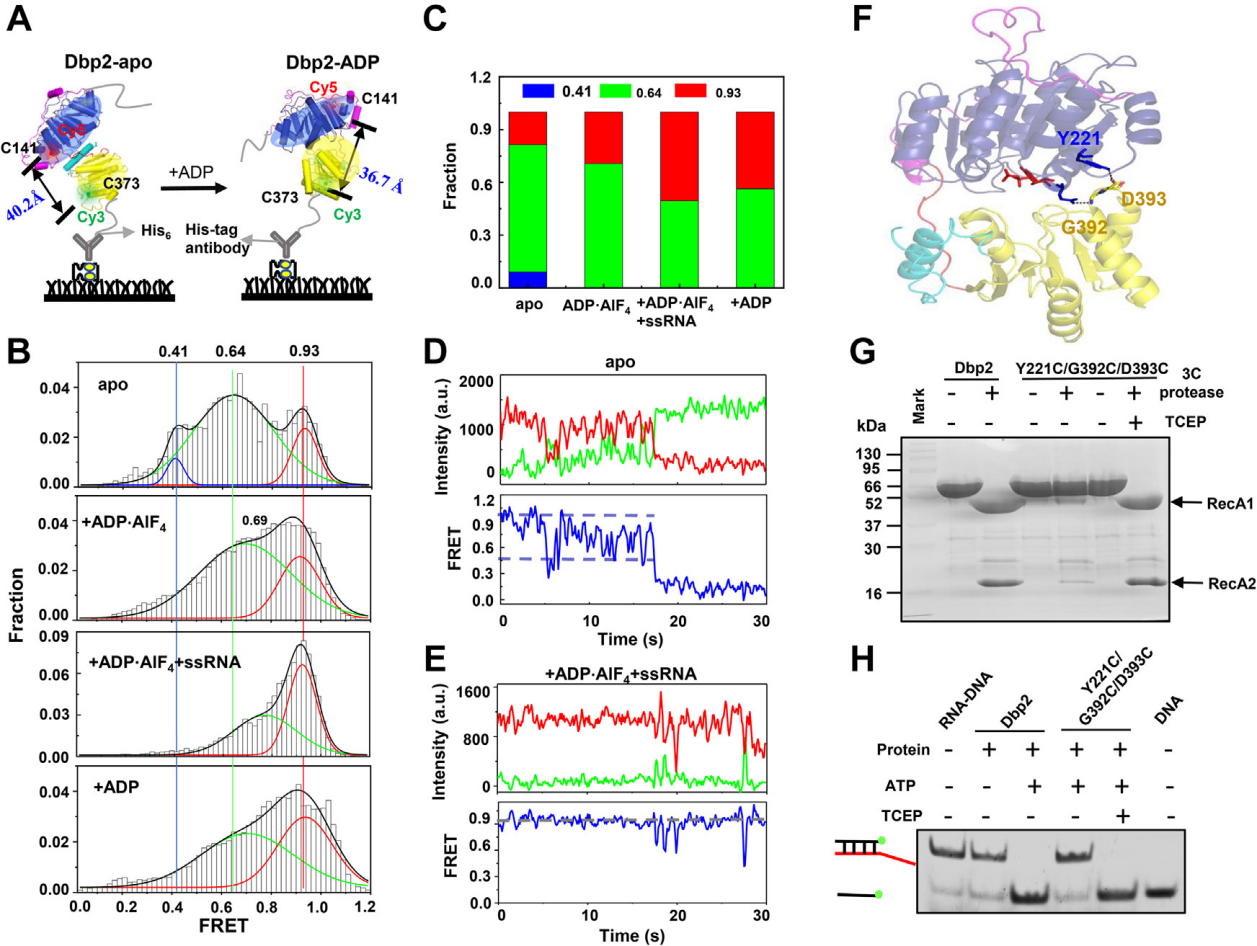
**Conformational changes of Dbp2 with bound and unbound nucleotides and ssRNA in solution.***A*, distance between C141 and C373 in the open and closed states, with Cy3 and Cy5 randomly labeled at the position of C141 and C373. Dbp2 with a His-tag was immobilized on PEG-passivated surface *via* a biotinylated His-tag antibody. *B*, FRET histograms of C141/C373 alone, with ADP·AlF_4_, with ADP·AlF_4_ and ssRNA, or with ADP. *C*, fraction of E_FRET_ ∼ 0.41 and E_FRET_ ∼ 0.64, and E_FRET_ ∼ 0.93 in the FRET histograms of Dbp2 alone, with ADP·AlF_4_, with ADP·AlF_4_ and ssRNA, with ADP. *D*, a single-molecule time trace and FRET efficiency for C141/C373 alone. *E*, single-molecule time trace and FRET efficiency of C141/C373 with ADP·AlF_4_ and ssRNA. *F*, residue Y221 in the RecA1 domain and residue G393 in the RecA2 domain form hydrogen bonds in the crystal structure of Dbp2-ADP. *G*, SDS-PAGE analyzed for disulfide bond formation in Y221C/G392C/D393C. The Y221C/G392C/D393C mutant forms a disulfide bond that locks in the RecA1 and RecA2 domains, leaving Dbp2 in a single state. *H*, the unwinding activity of Dbp2 and Y221C/G392C/D393C in the absence and the presence of TCEP. TCEP, Tris (2-carboxyethyl) phosphine.

To further elucidate the global conformational change of the helicase core in full-length Dbp2 upon binding of the nucleotide and ssRNA, we used single-molecule FRET (smFRET) to investigate the dynamic change of the two RecA-like domains in solution. Dbp2 contains four cysteines in the RecA1 domain and one cysteine in the RecA2 domain; we therefore mutated three cysteines to alanine in RecA1 (C168, C206, and C219) to obtain the C141/C373 mutant containing a single cysteine in each RecA-like domain. Finally, we obtained the double-labeled Dbp2 proteins C141/C373 carrying a different fluorescent dye in each RecA-like domain (Figs. 4*A* and S5*A*). The C141/C373 mutant showed WT-like binding and unwinding activities (Fig S5, *B* and *C*). Structural analysis of Dbp2 and DDX17 showed that the distances of the *C*_β_ positions of labeled-cysteine residues were approximately 40.2 to 47.2 Å apart in the open conformations and 35.8 to 36.7 Å apart in the closed conformations (Figs. 4*A* and S5*D*). In the smFRET experiment, the C141/C373 mutant was immobilized on the PEG-passivated surface *via* a biotinylated His-tag antibody.

We first monitored the FRET distribution of single Dbp2 molecules in the absence of substrate. The FRET histogram of unliganded Dbp2 shows three peaks of FRET efficiency (E_FRET_) corresponding to the open (E_FRET_ ∼ 0.41), semi-closed (E_FRET_ ∼ 0.64), and closed states (E_FRET_ ∼ 0.93) (Fig. 4, *B* and *C*, apo). The individual smFRET traces for Dbp2 showed that the FRET value fluctuated between ∼ 0.41 and ∼ 0.93 (Fig. 4*D*), indicating that the helicase core undergoes dynamic conformation changes in solution. This is consistent with crystal studies that report DEAD-box proteins in different conformations (28). We then examined the distributions of three FRET states of Dbp2 upon binding of ADP·AlF_4_, ADP·AlF_4_ and ssRNA, or ADP. As shown in Figure 4*B*, upon binding ADP·AlF_4_, the lowest FRET peak was still present and transited slightly to higher FRET peak (Fig. 4, *B* and *C*, ADP·AlF_4_). In contrast, Dbp2 in complex with ADP·AlF_4_ and ssRNA showed a remarkable increase in the E_FRET_ ∼ 0.93 peak (Fig. 4, *B*, *C* and *E*, ADP·AlF_4_ + ssRNA), which is consistent with the crystal structure of DDX17-ADP·BeF_3_^-^-U10 in the closed conformation. The FRET distribution of Dbp2-ADP was similar to that of Dbp2-ADP·AlF_4_ except for a decrease in the E_FRET_ ∼ 0.93 peak (Fig. 4, *B* and *C*, ADP). These results demonstrate that the helicase core of Dbp2 without ligands in the solution adopts different conformations and that the simultaneous presence of an ATP analog and ssRNA promotes a relatively closed conformation.

A previous study showed that DEAD-box protein unwinds nucleic acid duplexes using ATP-dependent conformational changes of the helicase core (29). To test whether the conformational change of the helicase core of Dbp2 is essential for unwinding activity, we used intramolecular crosslinking to lock the helicase core of Dbp2 in a single conformation and then detected for unwinding activity. In the crystal structure of Dbp2-ADP, residue Y221 of the RecA1 domain is close to residues G392 and D393 of the RecA2 domain (Fig. 4*F*); therefore, the spatial distance is in favor of an interdomain disulfide bridge formation between the RecA1 and RecA2 domains. To increase the chance of disulfide bridge formation, we constructed a three-cysteine- residues mutant Y221C/G392C/D393C to form intramolecular disulfide bonds. First, we replaced the sequence of the linker with that of the 3C protease recognition site to verify the formation of disulfide bonds in the Y221C/G392C/D393C mutant. If Y221C/G392C/D393C forms a disulfide bond, the position of the linker can be cleaved by a 3C protease but the disulfide bond still connects the helicase core; otherwise, the helicase core is split into two parts. SDS-PAGE analysis showed that WT Dbp2 could be cleaved by 3C protease either in the presence or absence of reducing agent tris (2-carboxyethyl) phosphine (TCEP), but the mutant could not be cleaved in the absence of TCEP (Fig. 4*G*), even upon increasing the amount of 3C protease (Fig S4*E*). However, Y221C/G392C/D393C was cleaved in the presence of TCEP, suggesting that mutant Y221C/G392C/D393C could form disulfide bonds that can be disrupted by TCEP (Fig S5*F*). Then, we measured the unwinding activity of Dbp2 and Y221C/G392C/D393C in the presence and absence of TCEP. As a control, Dbp2 and Y221C, G392C/D393C mutants displayed normal ATP-dependent unwinding activity in the absence of TCEP (Fig S5, *G* and *H*) but the unwinding activity of Y221C/G392C/D393C was negligible in the absence of 2 mM TCEP (Fig. 4*H*), suggesting that Dbp2 is inactive in a single conformation. These results provide direct evidence that the unwinding activity of the helicase core of Dbp2 depends on dynamic conformational changes.

### Inherently disordered N- and C-tails confer full helicase activities to the Dbp2 protein

**Figure 5:**
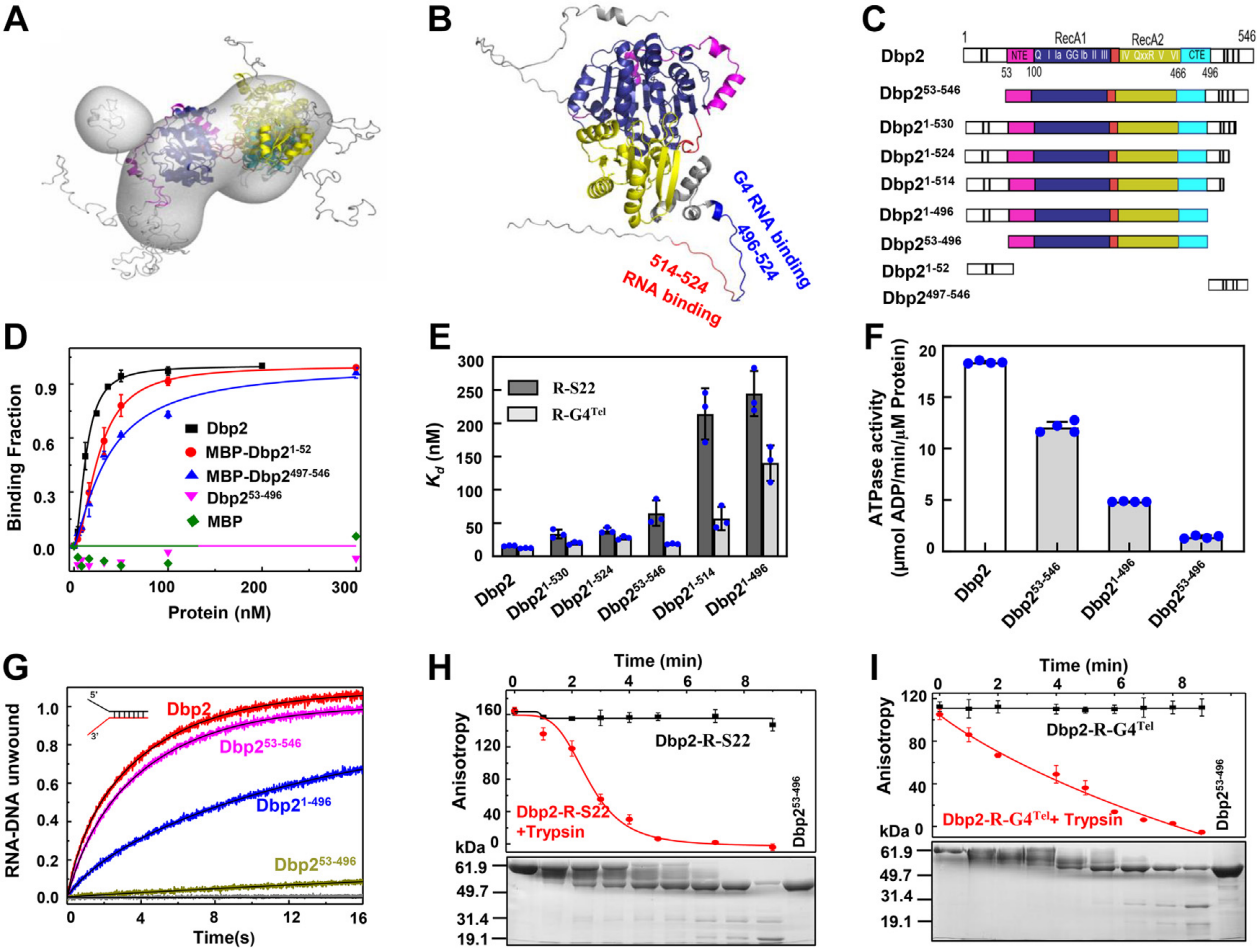
**The terminal domains of Dbp2 are responsible for binding, ATPase and unwinding activities.***A*, the modeled structure of the full-length Dbp2 is fitted in a SAXS *ab initio* envelope. The terminal tails are the disordered tails in *gray*. *B*, the structure of full-length Dbp2 is predicted by AlphaFold. The *red* and *blue regions* of the C-terminal tail show the critical ssRNA and G4 RNA-binding peptides. *C*, schematic representation of the domain of the Dbp2 truncation protein. *D*, binding affinities of Dbp2, MBP-Dbp2^1-52^, MBP-Dbp2^497-546^, Dbp2^53-496^, and MBP to R-G4^Tel^. The *K*_d_ value of Dbp2, MBP-Dbp2^1-52^, and MBP-Dbp2^497-546^ are 12.7 ± 0.5 nM，26.0 ± 1.5 nM, and 36.3 ± 5.1 nM, respectively. *E*, binding affinities of R-S22 and R-G4^Tel^ substrates to the Dbp2 truncated protein. *F*, ATPase activities of Dbp2, Dbp2^1-496^, Dbp2^53-546^, and Dbp2^53-496^ in the presence of R/D-D16. *G*, unwinding kinetics of a forked RNA/DNA duplex by Dbp2, Dbp2^1-496^, Dbp2^53-546^, Dbp2^53-496^, and Dbp2^118-466^ in the presence of ATP. The assays were performed as described in Materials and methods. *H* and *I*, values of the fluorescence anisotropy of Dbp2 bound to R-S22 or R-G4^Tel^ as a function of time to trypsin cleavage. Fragments of Dbp2 in complex with R-S22 or R-G4^Tel^ was examined by SDS-PAGE as a function of time to trypsin cleavage. Data show mean ± SD from three (*B*, *C*, *E*, *F* and *H*) and four (*I*) independent experiments. SAXS, Small-angle X-ray scattering.

To probe the spatial conformation of N- and C-tails relative to the full-length Dbp2 in solution, SAXS assays were performed. Fig S6*A* is the experimental SAXS profile of Dbp2. The Ensemble Optimization Method (30) analysis was carried out for Dbp2 by introducing flexibility in the N- and C-tails, and in the linker between the helicase core. An atomic model was finally obtained with χ^2^ of 1.62 (Fig. 5*A*). The full-length Dbp2 underwent dynamic changes in solution. The Kratky curve in Fig S6*B* further indicates that the full-length Dbp2 is disordered, because the protein with a compact spherical structure will generate a symmetrical curve. Consistently, the N- and C-tails of Dbp2 predicted by AlphaFold (https://alphafold.ebi.ac.uk/P24783) were also nonstructural (Fig. 5*B*). Inspection of the linear sequence of amino acids revealed that there are two and four conserved RGG motifs in the N- and C-tails, respectively. To further identify, which motifs play a crucial role in the regulation of helicase activity, the RGG tails were modified, resulting in six truncated versions, in which the RGG motif at the N-tail (Dbp2^53-546^ and Dbp2^53-496^) and the C-tail (Dbp2^1-496^ and Dbp2^53-496^) were deleted, respectively (Figs. 5*C* and S6*C*). The mutants (Dbp2^1-514^, Dbp2^1-524^, and Dbp2^1-530^) were the modified versions in which the RGG motifs in the C-tail were sequentially truncated, while maintaining N-tail integrity. Although we were unable to obtain the separate N- and C-tails (Dbp2^1-52^ and Dbp2^497-546^), they were fused with the MBP tag to purify.

Thus, the above mutants were characterized in parallel to the WT enzyme under the same experimental conditions. Characterizations of the WT and modified proteins revealed three surprising results. First, in the RNA binding assay, although the helicase core protein (Dbp2^53-496^) was totally inactive, the isolated N- and C-tails displayed RNA-binding activities comparable to the WT protein (Fig. 5*D*). Further, we found that the C-tail peptide 514 to 524 is critical to the binding of Dbp2 on R-S22 and peptide 496 to 524 is critical to the binding of R-G4^Tel^ (Fig. 5, *B* and *E*). Second, we examined the effects of N- and C-tails on the ATP-hydrolysis, unwinding, and annealing activities of Dbp2. Compared with the full-length Dbp2, the helicase core showed very poor ATP hydrolysis activity (Fig. 5*F*); meanwhile, no unwinding and annealing activities could be detected (Figs. 5*G* and S6*D*). In contrast, upon removal of the N-tail or C-tail, the protein retained its ATP hydrolysis and unwinding activities to varying degrees, but the C-tail was essential for the annealing activity (Fig. S6*D* and Table S6). Third, the most striking result was that the helicase core is entirely inactive without the assistance of the terminal tails (Fig. 5, *A–I*). To further confirm these results, a kinetic binding assay with labeled RNAs was carried out under equilibrium conditions using both anisotropy and SDS gel assays. In addition, parallel binding assays were performed under the same experimental conditions with and without proteolytic trypsin. Figure 5, *H* and *I* showed that Dbp2 binding activity gradually decreased with increasing time of trypsin cleavage.

Taken together, the N- and C-tails are critical to the RNA substrate binding, ATP hydrolysis, unwinding, and annealing activities of Dbp2. These results clearly indicate that the disordered terminal tails play an essential role in helicase activity: although the helicase core is inactive, the disordered terminal tails confer full helicase activity to the Dbp2 protein.

### The structurally disordered N- and C-tails become compact upon RNA binding and coordinate the helicase core domain

**Figure 6:**
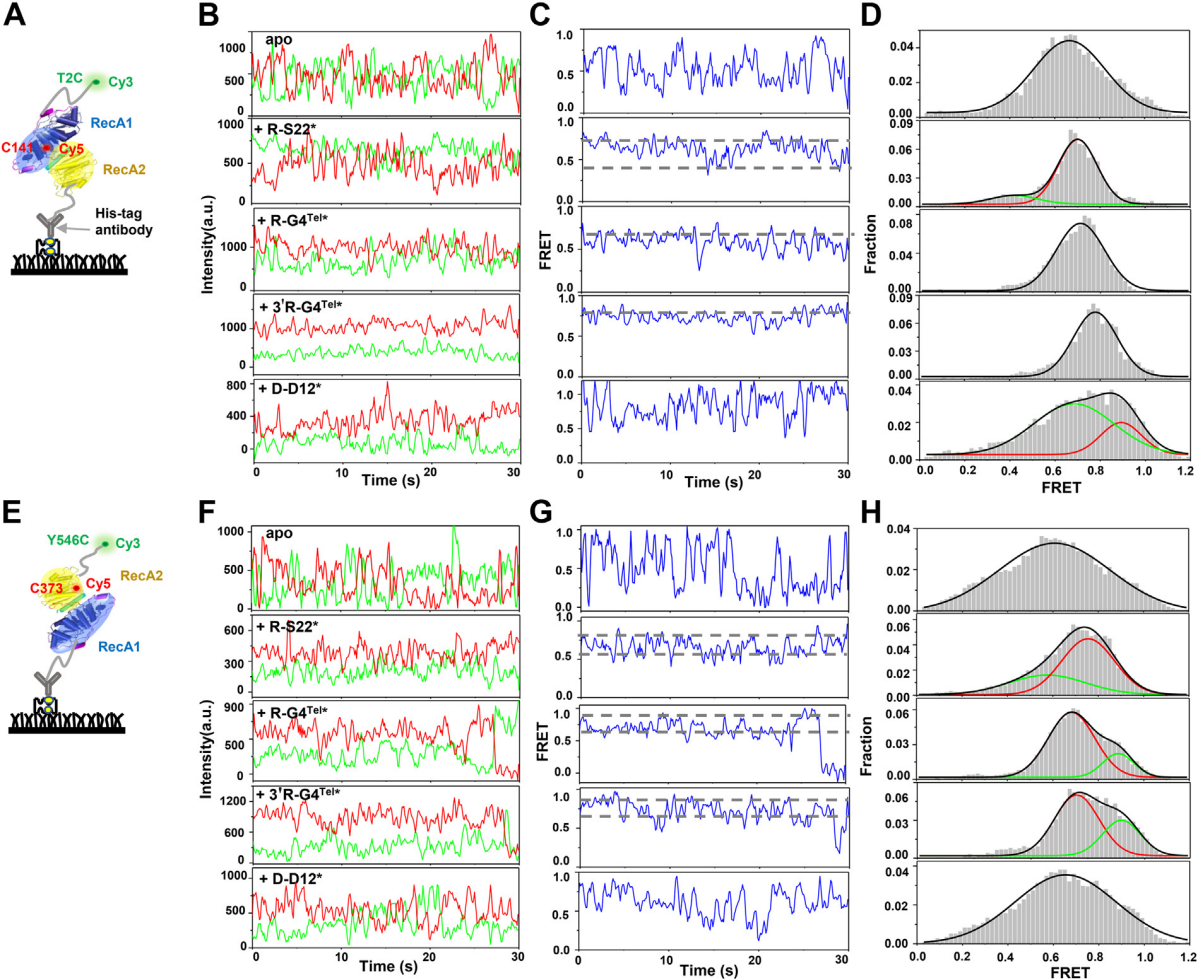
**Substrate binding experiments of N- and C-tails using smFRET experiment.***A*, schematic of the dye-labeled protein T2C/C141, with Cy3 and Cy5 randomly labeled at the T2C and C141 positions. *B* and *D*, donor and acceptor fluorescence trajectories (*B*), FRET efficiency trajectories (*C*) and FRET efficiency histograms (*D*) of T2C/C141 in the absence and presence of R-S22∗, R-G4^Tel^∗, 3′R-G4^Tel^∗ and D-D12∗. *E*, schematic of the dye-labeled protein C373/Y546C, with Cy3 and Cy5 randomly labeled at the C373 and Y546C positions. *F–H*, donor and acceptor fluorescence trajectories (*F*), FRET efficiency trajectories (*G*) and FRET efficiency histograms (*H*) of C373/Y546C in the absence and presence of R-S22∗, R-G4^Tel^∗, 3′R-G4^Tel^∗ and D-D12∗. smFRET, single-molecule FRET.

What is the potential molecular mechanism by which the “nonstructural N- and C-tails” confer full helicase activities to Dbp2? Using a computationally efficient approach that samples conformational space in a random manner (31), we found that the C_β_ distances between the N-terminal T2 and C141 of RecA1 is 25.9 Å and the C_β_ distances between the C-terminal Y546 and C373 of RecA2 is 40.8 Å; these distances are very suitable for use as pairs in FRET (Fig S6*E*) (32). Then, we constructed two mutants, T2C/C141 and C373/Y546C, which were stochastically labeled with Cy3 and Cy5 (Fig. 6, *A* and *E*). In T2C/C141, in addition to the mutation of threonine into cysteine at the second amino acid, all other cysteines were mutated into alanine. In C373/Y546C, in addition to tyrosine at amino acid 546 being mutated into cysteine, all other cysteines were mutated into alanine. Both mutants had normal helicase activity compared with WT Dbp2 (Fig S6*F*). The successfully fluorophore-labeled T2C/C141 or C373/Y546C was then immobilized on the surface *via* the biotinylated His-tag antibody.

We first measured the apo state of T2C/C141 and C373/Y546C. Both mutants showed a wide and flat FRET distribution with single peaks of E_FRET_ at 0.66 for T2C/C141 and E_FRET_ at 0.61 for C373/Y546C, respectively (Fig. 6, *D* and *H*, apo). In addition, the characteristic FRET traces of the two mutants show a high degree of fluctuations (Fig. 6, *B*, *C*, *F* and *G*, apo), which was similar to the helicase core conformation of Dbp2 in Figure 4*B*, indicating that the N- and C-tails are also highly dynamic in solution.

Next, we examined the FRET distribution of T2C/C141 and C373/Y546C in the presence of substrates. When R-S22∗, R-G4^Tel^∗, and 3′R-G4^Tel^∗ were added at saturated concentration to T2C/C141, the FRET distribution presented narrow peaks with higher E_FRET_ at ∼ 0.7, ∼ 0.71, and ∼ 0.78, respectively, indicating that the RNA substrate induced the N-tail to approach the RecA1 domain (Fig. 6, *B*–*D*). The fluctuation range of FRET traces also narrowed, suggesting that the structure shifted from a highly dynamic to a static state. This phenomenon is consistent with the single band in the nondenatured PAGE when Dbp2^1-496^ binds to the RNA substrate (Fig. S7, *A*–*D*). In contrast, after adding D-D12∗, the FRET histogram of T2C/C141 was similar to the apo state, consistent with the weak binding activity of Dbp2 to duplex DNA (Fig. 1*B*). After adding saturated concentrations of R-S22∗, R-G4^Tel^∗, and 3′R-G4^Tel^∗ to C373/Y546C, two peaks appeared in the FRET distribution (Fig. 6, *F*–*H*). The FRET level after binding with substrate also rapidly shifted between the two states (Fig. 6, *F*–*H*), indicating that the C-tail may exist in two different conformations when it is adjacent to the RecA2 domain. These observations are consistent with the two bands observed in the nondenatured PAGE when Dbp2^53-546^ was associated with the RNA substrate (Fig S7, *A*–*D*). As a control, the addition of D-D12∗ had little effect on C373/Y546C. Altogether, ssRNA, TelG4 RNA, and 3′TelG4 RNA bind to the N- and C-tails, which become compact and tether the substrates to the helicase core. As a result, the conformation of Dbp2 changes from a highly dynamic to a relatively static state after forming a complex with the substrates.

## Discussion

Dbp2, a DEAD-box protein in *S. cerevisiae*, has functions in regulating mRNP assembly (6), transcription initiation (5), and metabolic gene expression (33). Here, we not only reported the crystal structures of the Dbp2 catalytic core alone and in complex with ADP but also determined the function of the terminal tails. We solved the first crystal structure of Dbp2 to date, illustrating that the protein is rigid and in an open state. Similar conformations have also been found in Prp5 (34) and Prp28 (28). Interestingly, the addition of ADP changes the conformation of the helicase core from an open state to a nearly closed state, which is different from the ADP-bound structures of DDX19 (35), DDX21 (36) and Prp5 (34). Dbp2 bound and unbound ADP structures give important insights into the conformational changes between nucleotide-free and ADP-bound states. Similarly, smFRET experiments also captured the conformational changes of the helicase core in the absence and presence of nucleotides and ssRNA. Consequently, our study explains how the DEAD-box protein unfolds a duplex during a cycle of ATP-dependent conformational changes.

In many crystal structures of DEAD-box proteins, the NTEs and CTEs participate in structural plasticity at the terminal boundary of the helicase core. Moreover, they are essential for RNA binding, ATPase, and unwinding activities (25, 27, 35, 37, 38). The NTE is conserved between the Dbp2-apo and Dbp2-ADP structures and enwinds the exterior of the RecA1 domain, reaching near the RNA substrate binding area. In contrast, the CTE has two conformations: one in which Dbp2-apo is wedged between the helicase core and one in which the addition of ADP induces it to move out of the way of the ATP binding site. Mutational analyses also suggest that NTE and CTE are involved in regulating ATPase activity. Interestingly, the NTEs of Dbp2 and DDX17 simultaneously regulate ATPase and unwinding activities but they display different effects. Hence, knowing the structure and function of individual DEAD-box subfamily proteins provides a better understanding of the function of DEAD-box proteins *in vivo*.

As an RNA helicase, Dbp2 simultaneously possesses the ability of duplex disruption and formation; substrates with tails or blunt-ends are involved in the unwinding and annealing activities of Dbp2. Most DEAD-box proteins exclusively unfold RNA duplex substrates, but a few show both unwinding and annealing activity, such as Mss116p (39), CsdA (40) and Ded1 (41). There are several possible mechanisms that may mediate between the unwinding and annealing activities, such as protein oligomerization, ATP and ADP concentration, and the presence of an annealing domain (42). Here, upon truncation of the C-tail in Dbp2, annealing activity barely detectable, and unwinding activity was reduced by half. Hence, our results suggest that the C-tail of Dbp2 regulates the equilibrium between unwinding and annealing activities.

Due to the intrinsically disordered N- and C-terminal tails of DEAD-box protein, no structures of an intact full-length DEAD-box protein are available to date. Hence, we used SAXS and smFRET experiments to characterize the structural and conformational changes of full-length Dbp2 in solution. A previous study showed that the helicase core adopts a single conformation (43); our results showed that full-length Dbp2 adopts three flexible and dynamic conformations. Furthermore, we found that the N- and C-tails act as substrate binding sites and tether RNA substrates to the core domain. This result is consistent with previous studies on Mss116p and CsdA, in which C-tails can bind nonspecifically or specifically to RNA substrates and flexibly tether the substrates to the helicase core for the unwinding of neighboring duplexes (24, 44). Intrinsically disordered N- and C-tails in DEAD-box proteins contribute to mediating-specific protein and RNA interactions. Like other DEAD-box proteins, the N- tails of DDX43 (45) and LAF1 (46) contribute to RNA binding, the C-tails of DDX17 (25), DDX21 (47), DDX55 (48), and CsdA (44) are critical for RNA-binding activity, while the helicase core barely displays any RNA-binding activity. Hence, it is interesting to note that the nonstructured N- and C-tails likely act as tentacles to capture the RNA substrates and facilitate the unwinding, ATPase, and annealing activities of DEAD-box proteins. Our study demonstrates that nonstructured N- and C-tails display full Dbp2 activity, which may be a key to understanding the disordered terminal tails in DEAD-box protein function and their role in RNA metabolism.

## Experimental procedures

### Protein expression and purification

The complementary DNA fragments of Dbp2^1-546^, Dbp2^53-546^, Dbp2^1-496^, Dbp2^1-514^, Dbp2^1-524^, Dbp2^1-530^, and Dbp2^53-496^ were amplified by PCR and ligated into the pET21a HMT vector. The fused protein includes a His_6_-tag at the N terminus and a TEV protease cleavage site between Dbp2 and MBP. Protein expressions were done in *Escherichia coli* strain C2566H grown in LB medium at 37 °C to an A_600_ of 0.56 and induced with 0.2 mM IPTG at 18 °C overnight. Cells were collected and resuspended in buffer (50 mM Tris–HCl, pH 8.0, 500 mM NaCl, 10% Glycerol, 5 mM imidazole) and then lysed by sonication. After high-speed centrifugation, the supernatants were loaded on a Ni^2+^ charged immobilized metal ion affinity chromatography column (GE Healthcare) and washed with 1M NaCl to remove bound nucleic acids. The eluted proteins were cleaved using TEV proteases for 16 h. The proteins were further purified through an SP ion exchange chromatography (GE Healthcare), while Dbp2^53-496^ used Q ion exchange chromatography to purify. Purified proteins were concentrated and stored at −80 °C. All mutants were purified using the same protocol as Dbp2.

### Limited proteolysis and purification

Dbp2 proteins were incubated with trypsin at a range of concentrations (0, 0.00001, 0.0001, 0.001, 0.01, 0.1 mg/ml) in 50 μl reaction buffer (20 mM Tris–HCl pH 8.0, 600 mM NaCl, 10 mM CaCl_2_) at 16 °C for 1 h, then 1 mM ethylene diamine tetraacetic acid was added into reactions to stop the reactions. The SDS-PAGE analysis indicated that 0.0001 mg/ml of trypsin was the optimal enzyme concentration. Then, we tested the time to trypsin cleavage. Finally, we determined the reaction condition 0.0001 mg/ml trypsin to react for 2.5 h at 16 °C. For large-scale proteolysis, proteins, and trypsin were expanded proportionately. Proteolytic fragments were purified by Q ion exchange chromatography. The eluted proteins were combined and concentrated to 25 mg/ml for crystallization.

### Crystallization, data collection, and structure determination

Dbp2 proteolytic fragment was stored in the buffer (20 mM Hepes pH 7.0, 150 mM NaCl) for crystal screening. Crystals were grown using the sitting-drop vapor diffusion technique at 20 °C. The helicase core of Dbp2 was diluted to 8 mg/ml and in complex with 20 mM ADP. Crystals of the helicase core of Dbp2 were grown in a reservoir solution containing 0.12 M monosaccharides, 1 M sodium Hepes; 3-morpholinopropane-1-sulfonic (acid) pH 7.5, 19% w/v PEG 500MME PEG 20000. Crystals of Dbp2 helicase core in complex with ADP and 16 nt polyGT ssDNA were grown in reservoir solution containing 2.02 M (NH4)_2_SO_4_ and 100 mM Tris pH 7.5. The crystals are acquired after 1 to 2 days.

For data collection, 20% ethylene glycol was added to the reservoir solution and the crystals were immediately mounted and flash cooled in liquid N_2_. X-ray diffraction data were collected on beamline BL18U1 beamline at Shanghai Synchrotron Radiation Facility (SSRF) and were processed using the XDS package (http://xds.mpimf-heidelberg.mpg.de/) (49). The Dbp2 structure was solved by molecular replacement. Phenix was used for refined (50) and Coot was used for model building (51). For twinning problems of Dbp2-ADP-ssDNA crystal, we made a lot of efforts and optimally selected P2_1_2_1_2_1_ space group. Crystals had unit cell 168.84 168.84 145.93 90.0 90.0 90.0 and could be apparently indexed in space group P4_3_2_1_2 with three molecules in the asymmetric unit. Though data collection statistics were good, solving the structure in this space group by molecular replacement are impossible with no solution found. Apparent P4_3_2_1_2 could be due to perfect merohedral twinning and possible space groups can be P4_3_ or P2_1_2_1_2_1_ as described in (52). We tried subgroups P4_3_ with twin law h, -k, -l, and P2_1_2_1_2_1_ with twin law -h, l, k and six molecules in the asymmetric unit for both cases. Twin fraction is 0.43 as calculated by Britton analyses. P2_1_2_1_2_1_ gives better refinement statistics and we choose this space group. The detailed structure determination and refinement statistics are shown in Table S2.

### Small-angle X-ray scattering

The full-length Dbp2 was prepared at 5.0 mg/ml in 25 mM Hepes pH 7.5, 500 mM NaCl, and 20% glycerol buffer. SAXS data were collected at the BL19U2 beamline of the Shanghai Synchrotron Radiation Facility. The SAXS data for Dbp2^53-496^ with ADP was collected at beamline SWING (SOLEIL Synchrotron, Saint-Aubin). The scattering processing was measured as described (53). The SAXS data were analyzed using ATSAS (54) and model building was done with Ensemble Optimization Method (30). All SAXS data are shown in Table S3.

### CD spectroscopy

CD spectra measurements were performed using a spectropolarimeter (Applied Photophysics). Purified Dbp2, NTE, and CTE mutants contained N-terminal MBP tag dialyzed in sodium phosphate buffer (Na_2_HPO_4_/NaH_2_PO_4_, pH 7.5) at 25 °C with a protein concentration of 3 μM. CD spectra were collected in the wavelength range of 190 to 260 nm. CD results were analyzed using the CAPITO software (https://capito.software/) (55).

### ATPase activity assay

The ATPase activity of Dbp2 and mutants was measured using an ATPase/GTPase activity assay kit (Sigma MAK113). Briefly, Dbp2 and several mutants were diluted at a final concentration of 0.1 μM in assay buffer (25 mM Tris–HCl pH 7.5, 30 mM KCl, 2 mM MgCl_2_). Dbp2 and several deletion constructions were diluted in the assay buffer at a final concentration of 0.5 μM. The assay reaction mixture contained 0.75 μM 16 bp RNA-DNA hybrid duplex, 1.5 mM ATP, and assay buffer was added to bring the volume to 20 μl. The reactions were processed at 24 °C for 15 min and were quenched by adding 100 μl regent. The absorbance was read out at 620 nm. The standard curve equations were used to calculate the free phosphate.

### Fluorescent labeling

The purified C141/C373 and T2C/C141 proteins contain a His_6_-tag at the C terminus and the C373/Y546C protein contains a His_6_-tag at the N terminus. Before labeling, mutant proteins were incubated with 1 mM TCEP for 30 min at room temperature to reduce disulfide bonds. For fluorescent labeling, the double-Cys mutants were randomly labeled by sequentially adding Cy3- and Cy5-maleimide fluorophore (Lumiprobe). The dye and protein molar ratio are 5: 1. The reaction proceeded overnight at the room temperature while stirring in the dark. Free Cy3 and Cy5 fluorophore was removed by Ni affinity chromatography. The fluorescent-labeled proteins were eluted from Ni affinity chromatography. The 12% SDS-PAGE detected the labeled extent of proteins, the labeling efficiency was calculated by gel analysis and it was more than 80%. The concentrations of labeled proteins were measured by Thermo Fisher Scientific Nanodrop 2000c. Labeled proteins were stored at −80 °C in 50 mM Tris–HCl, pH 8.0, 1 mM DTT, 600 mM NaCl, and 20% (vol/vol) glycerol.

### smFRET measurements

smFRET assay was performed with a home-built objective-type total-internal-reflection microscopy (56). In short, 50 nM C141/C373 was added to the chamber and allowed to be immobilized for 10 min, then free C141/C373 molecule was removed by wash buffer 25 mM Tris–HCl pH 7.5, 30 mM KCl, 2 mM MgCl_2_, and an oxygen scavenging system (0.8% D-glucose, 1 mg/ml glucose oxidase, 0.4 mg/ml catalase, and 1 mM Trolox). Imaging was initiated after C141/C373. Single-molecule measurements were performed using an exposure time of 100 ms. FRET analysis was referred to describe previously (57). The smFRET experiments of T2C/C141 and C373/Y546C were performed in similar process.

### Stopped-flow FRET measurements

A stopped-flow FRET assay was used to measure the unwinding kinetic rate constants of Dbp2 and its variants. The unwinding experiments were performed as described (58). Briefly, the reaction buffer contains 25 mM Tris–HCl (pH 7.5), 30 mM KCl, and 2 mM MgCl_2_. 25 nM Dbp2 and 8 nM substrates (Table S7) were mixed in the reaction buffer. After incubation for 5 min at 25 °C. The reaction was initiated by adding 2 mM ATP. Unwinding kinetic rate constants and amplitude were analyzed as described (59).

The annealing experiment was measured using a stopped-flow FRET assay. The whole operation is done in a similar way to the unwinding experiment. The experiments were completed at 25 °C in a buffer of 25 mM Tris–HCl (pH 7.5), 30 mM KCl, and 2 mM MgCl_2_. Two complementary strands containing one of them were mixed with 25 nM Dbp2 and another strand was added to initiate the reaction. The data analysis refers to describe previously (60).

### Electrophoretic mobility shift assay

Fam-labeled R-S22, R-G4^Tel^, 3′R-G4^Tel^, and 5′R-G4^Tel^ were mixed with varying amounts of proteins in the binding buffer containing 25 mM Tris–HCl (pH 7.5), 30 mM KCl, 2 mM MgCl_2_. The complexes were separated by native PAGE and visualized using a ChemiDoc MP (BIO-RAD).

### Nucleic acid binding assay

A fluorescence anisotropy assay was used to determine the apparent dissociation constants of the proteins under the equilibrium nucleic acid binding conditions. Protein concentration-dependent changes in fluorescence anisotropy were measured with fluorescein-labeled nucleic acid by fluorescence polarization assay using an Infinite F200 instrument (TECAN). Varying amounts of protein were added to a 150 μl aliquot of buffer (25 mM Tris–HCl, pH 7.5, 20 mM NaCl/30 mM KCl, 2 mM MgCl_2_, and 1 mM DTT) containing 5 nM fluorescein-labeled nucleic acid (Table S7). Protein and substrate were incubated at 25 °C for 5 min. After 5 min, the steady-state fluorescence anisotropy (*r*) was measured. Calculation of the equilibrium dissociation constant as described (61).

## Data availability

All data are contained within the article. Data necessary to validate protein structure determination and modeling can be obtained in the Protein Data Bank under the following accession number: Dbp2-apo: 8ARK; Dbp2-ADP: 8ARP.

## Supporting information

This article contains supporting information.

## Conflict of interest

The authors declare that they have no conflicts of interest with the contents of this article.

## References

[1] Gorbalenya A.E., Koonin E.V. (1993). Helicases: amino acid sequence comparisons and structure-function relationships. Curr. Opin. Struct. Biol..

[2] Jarmoskaite I., Russell R. (2014). RNA helicase proteins as chaperones and remodelers. Annu. Rev. Biochem..

[3] Ma W.K., Paudel B.P., Xing Z., Sabath I.G., Rueda D., Tran E.J. (2016). Recruitment, duplex unwinding and protein-mediated inhibition of the dead-box RNA helicase Dbp2 at actively transcribed chromatin. J. Mol. Biol..

[4] Lai Y.H., Choudhary K., Cloutier S.C., Xing Z., Aviran S., Tran E.J. (2019). Genome-wide discovery of DEAD-box RNA helicase targets reveals RNA structural remodeling in transcription termination. Genetics.

[5] Cloutier S.C., Wang S., Ma W.K., Al Husini N., Dhoondia Z., Ansari A. (2016). Regulated formation of lncRNA-DNA hybrids enables faster transcriptional induction and environmental adaptation. Mol. Cell.

[6] Ma W.K., Cloutier S.C., Tran E.J. (2013). The DEAD-box protein Dbp2 functions with the RNA-binding protein Yra1 to promote mRNP assembly. J. Mol. Biol..

[7] Yu Z., Mersaoui S.Y., Guitton-Sert L., Coulombe Y., Song J., Masson J.Y. (2020). DDX5 resolves R-loops at DNA double-strand breaks to promote DNA repair and avoid chromosomal deletions. NAR Cancer.

[8] Gao J., Byrd A.K., Zybailov B.L., Marecki J.C., Guderyon M.J., Edwards A.D. (2019). DEAD-box RNA helicases Dbp2, Ded1 and Mss116 bind to G-quadruplex nucleic acids and destabilize G-quadruplex RNA. Chem. Commun. (Camb).

[9] Metifiot M., Amrane S., Litvak S., Andreola M.L. (2014). G-Quadruplexes in viruses: function and potential therapeutic applications. Nucl. Acids Res..

[10] Kovalev N., Barajas D., Nagy P.D. (2012). Similar roles for yeast Dbp2 and Arabidopsis RH20 DEAD-box RNA helicases to Ded1 helicase in tombusvirus plus-strand synthesis. Virology.

[11] Hashemi V., Masjedi A., Hazhir-Karzar B., Tanomand A., Shotorbani S.S., Hojjat-Farsangi M. (2019). The role of DEAD-box RNA helicase p68 (DDX5) in the development and treatment of breast cancer. J. Cell Physiol..

[12] Shin S., Rossow K.L., Grande J.P., Janknecht R. (2007). Involvement of RNA helicases p68 and p72 in colon cancer. Cancer Res..

[13] Clark E.L., Coulson A., Dalgliesh C., Rajan P., Nicol S.M., Fleming S. (2008). The RNA helicase p68 is a novel androgen receptor coactivator involved in splicing and is overexpressed in prostate cancer. Cancer Res..

[14] Wang Z., Luo Z., Zhou L., Li X., Jiang T., Fu E. (2015). DDX5 promotes proliferation and tumorigenesis of non-small-cell lung cancer cells by activating beta-catenin signaling pathway. Cancer Sci..

[15] Du C., Li D.Q., Li N., Chen L., Li S.S., Yang Y. (2017). DDX5 promotes gastric cancer cell proliferation *in vitro* and *in vivo* through mTOR signaling pathway. Sci. Rep..

[16] Lin S., Tian L., Shen H., Gu Y., Li J.L., Chen Z. (2013). DDX5 is a positive regulator of oncogenic NOTCH1 signaling in T cell acute lymphoblastic leukemia. Oncogene.

[17] Xing Z., Wang S., Tran E.J. (2017). Characterization of the mammalian DEAD-box protein DDX5 reveals functional conservation with *S. Cerevisiae* Ortholog Dbp2 transcriptional control glucose metabolism. RNA.

[18] Sengoku T., Nureki O., Nakamura A., Kobayashi S., Yokoyama S. (2006). Structural basis for RNA unwinding by the DEAD-box protein Drosophila Vasa. Cell.

[19] Banroques J., Cordin O., Doere M., Linder P., Tanner N.K. (2011). Analyses of the functional regions of DEAD-box RNA "helicases" with deletion and chimera constructs tested *in vivo* and *in vitro*. J. Mol. Biol..

[20] Gilman B., Tijerina P., Russell R. (2017). Distinct RNA-unwinding mechanisms of DEAD-box and DEAH-box RNA helicase proteins in remodeling structured RNAs and RNPs. Biochem. Soc. Trans..

[21] Hondele M., Sachdev R., Heinrich S., Wang J., Vallotton P., Fontoura B.M.A. (2019). DEAD-box ATPases are global regulators of phase-separated organelles. Nature.

[22] Thandapani P., O'Connor T.R., Bailey T.L., Richard S. (2013). Defining the RGG/RG motif. Mol. Cell.

[23] Yan K.K., Obi I., Sabouri N. (2021). The RGG domain in the C-terminus of the DEAD box helicases Dbp2 and Ded1 is necessary for G-quadruplex destabilization. Nucl. Acids Res..

[24] Mallam A.L., Jarmoskaite I., Tijerina P., Del Campo M., Seifert S., Guo L. (2011). Solution structures of DEAD-box RNA chaperones reveal conformational changes and nucleic acid tethering by a basic tail. Proc. Natl. Acad. Sci. U. S. A..

[25] Ngo T.D., Partin A.C., Nam Y. (2019). RNA specificity and autoregulation of DDX17, a modulator of MicroRNA biogenesis. Cell Rep..

[26] Xing Z., Ma W.K., Tran E.J. (2019). The DDX5/Dbp2 subfamily of DEAD-box RNA helicases. Wiley Interdiscip Rev RNA.

[27] Dutta S., Gupta G., Choi Y.W., Kotaka M., Fielding B.C., Song J. (2012). The variable N-terminal region of DDX5 contains structural elements and auto-inhibits its interaction with NS5B of hepatitis C virus. Biochem. J..

[28] Mohlmann S., Mathew R., Neumann P., Schmitt A., Luhrmann R., Ficner R. (2014). Structural and functional analysis of the human spliceosomal DEAD-box helicase Prp28. Acta Crystallogr. D Biol. Crystallogr..

[29] Yingfeng Chen J.P.P., Tijerina P., Campo M.D., Lambowitz A.M., Russell R. (2008). DEAD-box proteins can completely separate an RNA duplex using a single ATP. Proc. Natl. Acad. Sci. U. S. A..

[30] Tria G., Mertens H.D., Kachala M., Svergun D.I. (2015). Advanced ensemble modelling of flexible macromolecules using X-ray solution scattering. IUCrJ.

[31] Yuan F., Griffin L., Phelps L., Buschmann V., Weston K., Greenbaum N.L. (2007). Use of a novel Förster resonance energy transfer method to identify locations of site-bound metal ions in the U2-U6 snRNA complex. Nucl. Acids Res..

[32] Dai Y.X., Chen W.F., Liu N.N., Teng F.Y., Guo H.L., Hou X.M. (2021). Structural and functional studies of SF1B Pif1 from Thermus oshimai reveal dimerization-induced helicase inhibition. Nucl. Acids Res..

[33] Wang S., Xing Z., Pascuzzi P.E., Tran E.J. (2017). Metabolic adaptation to nutrients involves coregulation of gene expression by the RNA helicase Dbp2 and the Cyc8 corepressor in Saccharomyces cerevisiae. G3 (Bethesda).

[34] Zhang Z.M., Yang F., Zhang J., Tang Q., Li J., Gu J. (2013). Crystal structure of Prp5p reveals interdomain interactions that impact spliceosome assembly. Cell Rep..

[35] Collins R., Karlberg T., Lehtio L., Schutz P., van den Berg S., Dahlgren L.G. (2009). The DEXD/H-box RNA helicase DDX19 is regulated by an α-helical switch. J. Biol. Chem..

[36] Chen Z., Li Z., Hu X., Xie F., Kuang S., Zhan B. (2020). Structural basis of human helicase DDX21 in RNA binding, unwinding, and antiviral signal activation. Adv. Sci. (Weinh).

[37] Mohr G., Del Campo M., Turner K.G., Gilman B., Wolf R.Z., Lambowitz A.M. (2011). High-throughput genetic identification of functionally important regions of the yeast DEAD-box protein Mss116p. J. Mol. Biol..

[38] Floor S.N., Condon K.J., Sharma D., Jankowsky E., Doudna J.A. (2016). Autoinhibitory interdomain interactions and subfamily-specific extensions redefine the catalytic core of the human DEAD-box protein DDX3. J. Biol. Chem..

[39] Halls C., Mohr S., Campo M.D., Yang Q., Jankowsky E., Lambowitz A.M. (2007). Involvement of DEAD-box proteins in group I and II intron splicing. Biochemical characterization of Mss116p, ATP-hydrolysis-dependent and -independent mechanisms, and general RNA chaperone activity. J. Mol. Biol..

[40] Stampfl S., Doetsch M., Beich-Frandsen M., Schroeder R. (2013). Characterization of the kinetics of RNA annealing and strand displacement activities of the E. coli DEAD-box helicase CsdA. RNA Biol..

[41] Sharma D., Putnam A.A., Jankowsky E. (2017). Biochemical differences and similarities between the DEAD-box helicase orthologs DDX3X and Ded1p. J. Mol. Biol..

[42] Ramanagoudr-Bhojappa R., Byrd A.K., Dahl C., Raney K.D. (2014). Yeast Pif1 accelerates annealing of complementary DNA strands. Biochemistry.

[43] Ramachandran A., Summerville L., Learn B.A., DeBell L., Bailey S. (2008). Cooperative binding of ATP and RNA induces a closed conformation in a DEAD box RNA helicase. Proc. Natl. Acad. Sci. U. S. A..

[44] Xu L., Wang L., Peng J., Li F., Wu L., Zhang B. (2017). Insights into the structure of dimeric RNA helicase CsdA and indispensable role of its C-terminal regions. Structure.

[45] Yadav M., Singh R.S., Hogan D., Vidhyasagar V., Yang S., Chung I.Y.W. (2021). The KH domain facilitates the substrate specificity and unwinding processivity of DDX43 helicase. J. Biol. Chem..

[46] Elbaum-Garfinkle S., Kim Y., Szczepaniak K., Chen C.C., Eckmann C.R., Myong S. (2015). The disordered P granule protein LAF-1 drives phase separation into droplets with tunable viscosity and dynamics. Proc. Natl. Acad. Sci. U. S. A..

[47] Marcaida M.J., Kauzlaric A., Duperrex A., Sulzle J., Moncrieffe M.C., Adebajo D. (2020). The human RNA helicase DDX21 presents a dimerization interface necessary for helicase activity. iScience.

[48] Choudhury P., Kretschmer J., Hackert P., Bohnsack K.E., Bohnsack M.T. (2021). The DExD box ATPase DDX55 is recruited to domain IV of the 28S ribosomal RNA by its C-terminal region. RNA Biol..

[49] Kabsch W. (2010). Xds. Acta Crystallogr. D Biol. Crystallogr..

[50] Adams P.D., Afonine P.V., Bunkoczi G., Chen V.B., Davis I.W., Echols N. (2010). Phenix: a comprehensive python-based system for macromolecular structure solution. Acta Crystallogr. D Biol. Crystallogr..

[51] Emsley P., Lohkamp B., Scott W.G., Cowtan K. (2010). Features and development of Coot. Acta Crystallogr. D Biol. Crystallogr..

[52] Kim W.M., Sigalov A.B., Stern L.J. (2010). Pseudo-merohedral twinning and noncrystallographic symmetry in orthorhombic crystals of SIVmac239 Nef core domain bound to different-length TCRzeta fragments. Acta Crystallogr. D Biol. Crystallogr..

[53] Zhai L.T., Rety S., Chen W.F., Song Z.Y., Auguin D., Sun B. (2021). Crystal structures of N-terminally truncated telomerase reverse transcriptase from fungidouble dagger. Nucl. Acids Res..

[54] Petoukhov M.V., Franke D., Shkumatov A.V., Tria G., Kikhney A.G., Gajda M. (2012). New developments in the ATSAS program package for small-angle scattering data analysis. J. Appl. Crystallogr..

[55] Wiedemann C., Bellstedt P., Gorlach M. (2013). CAPITO--a web server-based analysis and plotting tool for circular dichroism data. Bioinformatics.

[56] Wu W.Q., Hou X.M., Li M., Dou S.X., Xi X.G. (2015). BLM unfolds G-quadruplexes in different structural environments through different mechanisms. Nucl. Acids Res..

[57] Hou X.M., Fu Y.B., Wu W.Q., Wang L., Teng F.Y., Xie P. (2017). Involvement of G-triplex and G-hairpin in the multi-pathway folding of human telomeric G-quadruplex. Nucl. Acids Res..

[58] Liu N.N., Duan X.L., Ai X., Yang Y.T., Li M., Dou S.X. (2015). The Bacteroides sp. 3_1_23 Pif1 protein is a multifunctional helicase. Nucl. Acids Res..

[59] Liu N.N., Song Z.Y., Guo H.L., Yin H., Chen W.F., Dai Y.X. (2021). Endogenous Bos taurus RECQL is predominantly monomeric and more active than oligomers. Cell Rep..

[60] Dou S.X., Xi X.G. (2010). Fluorometric assays for characterizing DNA helicases. Methods.

[61] Chen W.F., Rety S., Guo H.L., Dai Y.X., Wu W.Q., Liu N.N. (2018). Molecular mechanistic insights into Drosophila DHX36-mediated G-quadruplex unfolding: a structure-based model. Structure.

