# Supplementary material for: Nonstructural N- and C-tails of Dbp2 confer the protein full helicase activities
Source: J Biol Chem. 2023 Mar 8;299(5):104592. doi: 10.1016/j.jbc.2023.104592 (PMC10193013; doi:10.1016/j.jbc.2023.104592)
Supplement: Supporting information [file mmc1.docx]

**Supplemental information**

**Nonstructural N- and C- tails of Dbp2 confer the protein full helicase activities**

Qin-Xia Song, Na-Nv Liu, Zhao-Xia Liu, Ying-Zi Zhang, Stephane Rety, Xi-Miao Hou and Xu-Guang Xi

Correspondence author: correspondence; Email: [xxi01@ens-cachan.fr](mailto:xxi01@ens-cachan.fr)

**
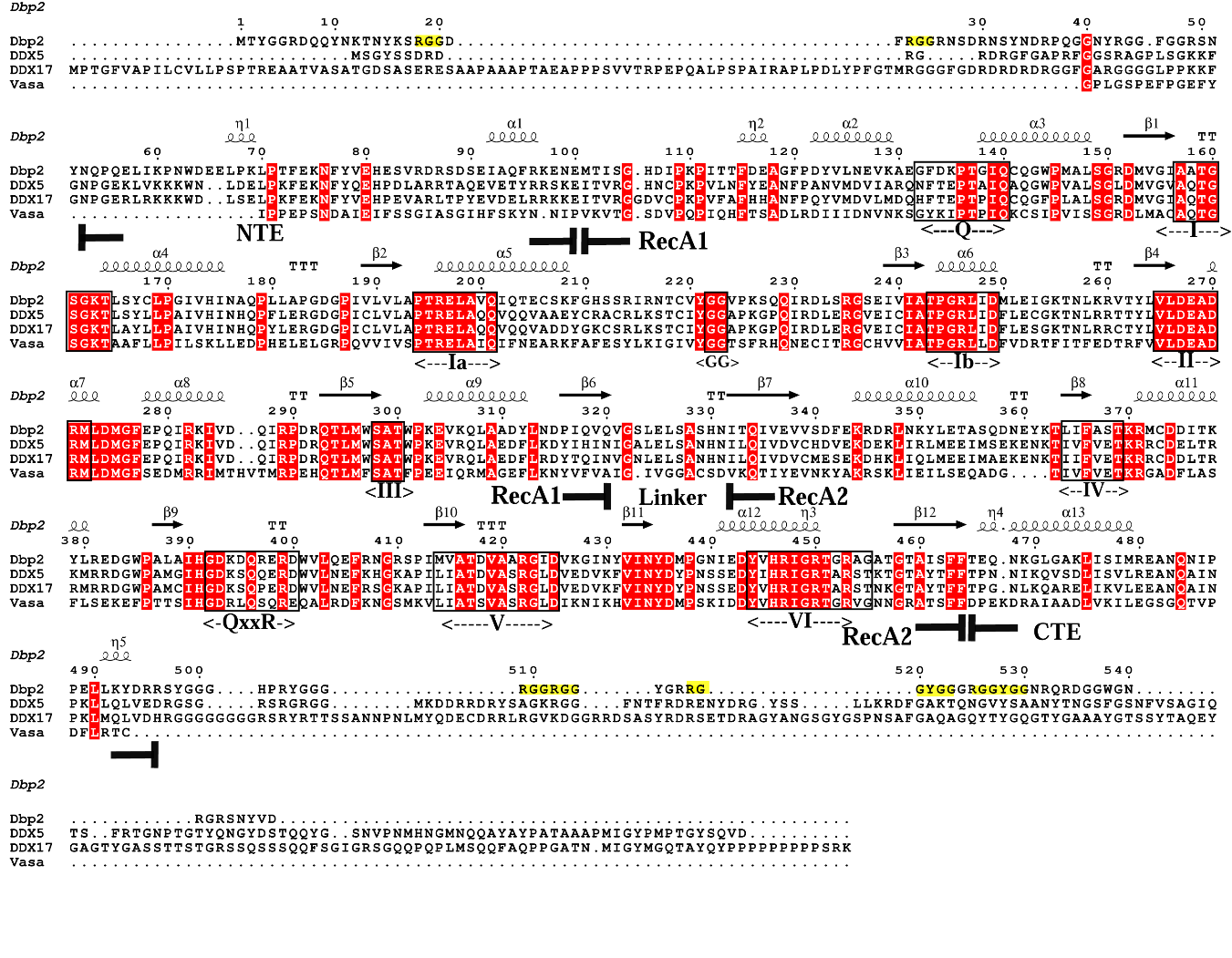
**

**Figure S1.** Sequence alignment of *Saccharomyces cerevisiae* Dbp2, *Homo* *sapiens* DDX5, *Homo* *sapiens* DDX17, *Drosophila melanogaster* Vasa. The black-bordered box indicates conserved sequence motifs. White letters show identical amino acids, and black letters show variant amino acids.


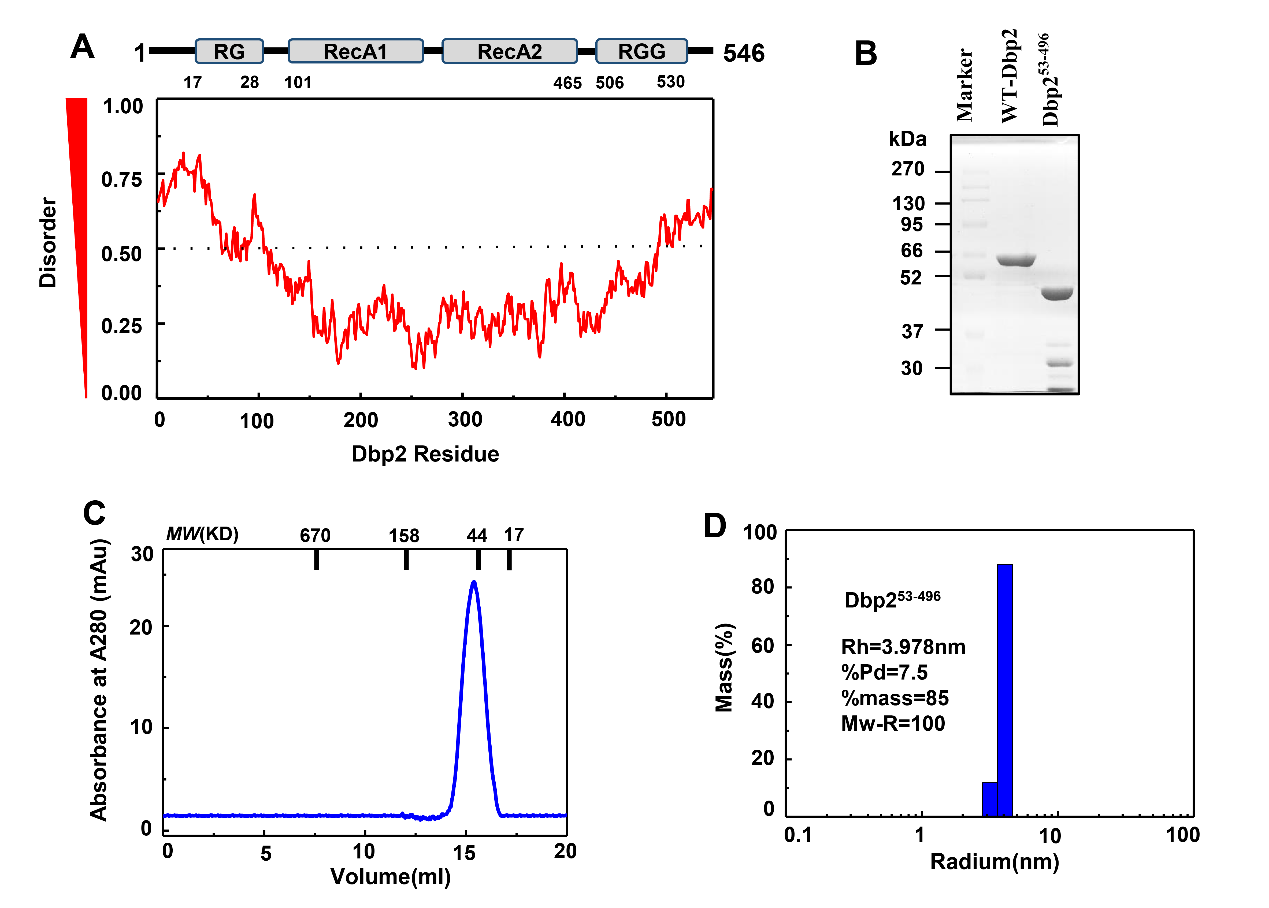


**Figure S2.** Purification and aggregate state analysis of Dbp2^53-496^. **A**, disorder tendency analysis of Dbp2. The horizontal axis is the amino acid position and the vertical axis is the disordering tendency. A disorder tendency value less than 0.5 indicates protein sequence is in order and is favorable to crystallization. **B**, SDS-PAGE analysis of purified Dbp2 and Dbp2^53-496^. **C**, size exclusion chromatography analysis of Dbp2^53-496^ by superdex 200 10/300 GL. **D**, dynamic light scattering experiment of Dbp2^53-496^.

|  | **MR Difference map Fo-Fc 2.5σ** | **Refined final map 2Fo-Fc 1σ** | **Composite omit map 2Fo-Fc 2σ** |
| --- | --- | --- | --- |
| **Chain A** | 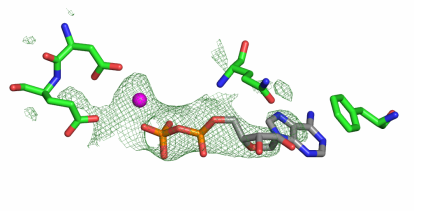 | 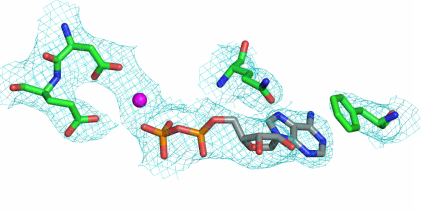  **ADP**  **texte ici**  **F133 texte ici**  **E268 texte ici**  **Mg**  **texte ici**  **E267 texte ici**  **Q140**  **texte ici** | 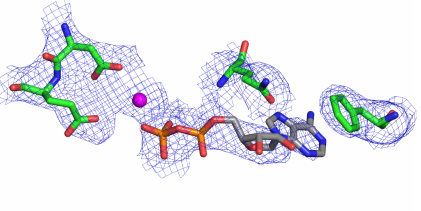 |
| **Chain B** | 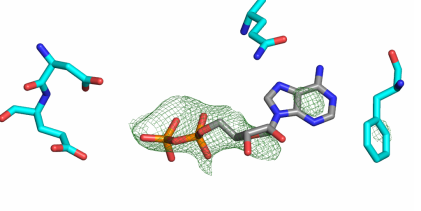 | 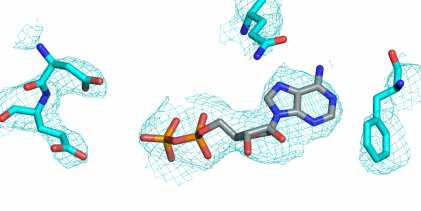 | 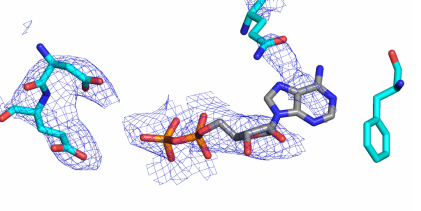 |
| **Chain C** | 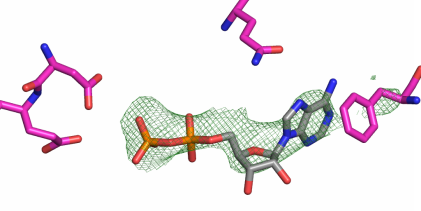 | 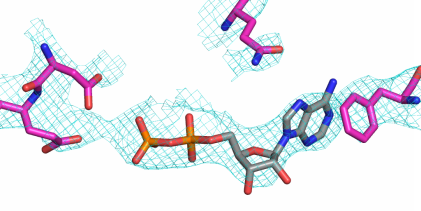 | 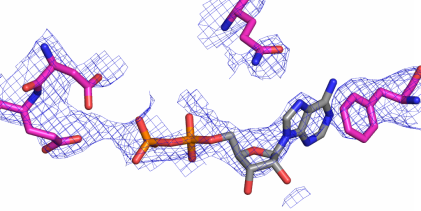 |
| **Chain D** | 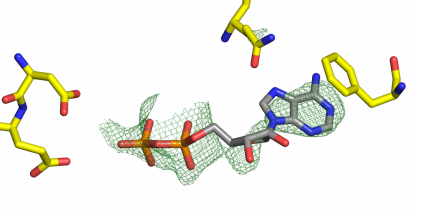 | 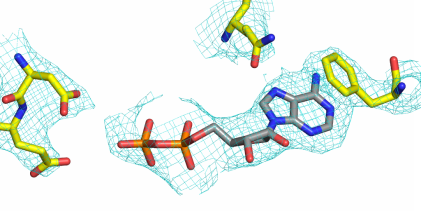 | 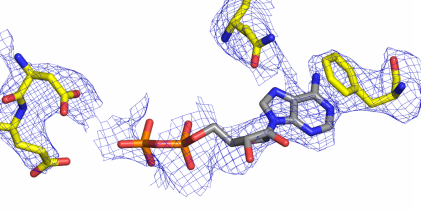 |
| **Chain E** | 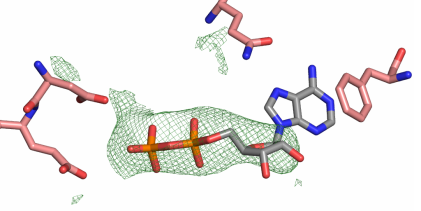 | 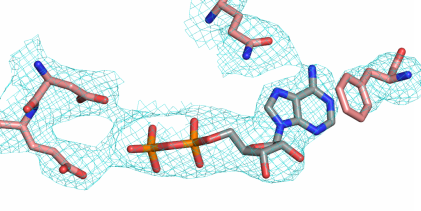 | 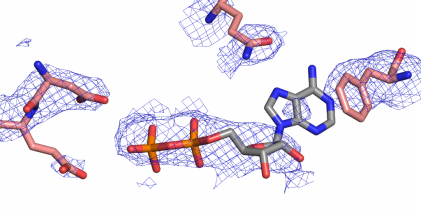 |
| **Chain F** | 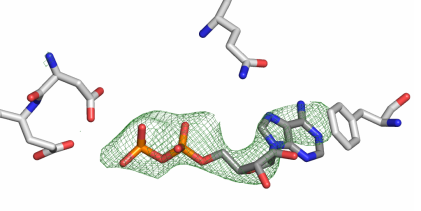 | 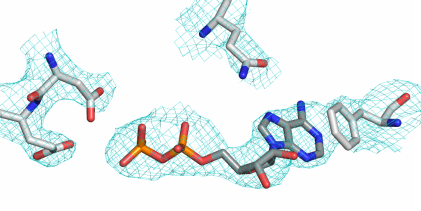 | 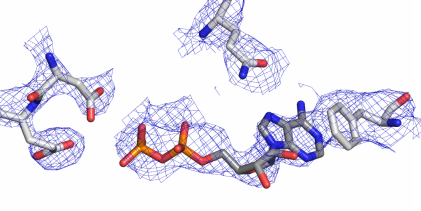 |

**Figure S3**. Molecular replacement Fo-Fc difference map, final refined 2Fo-Fc map and composite omit map around ADP of the six molecules in the asymmetric unit.


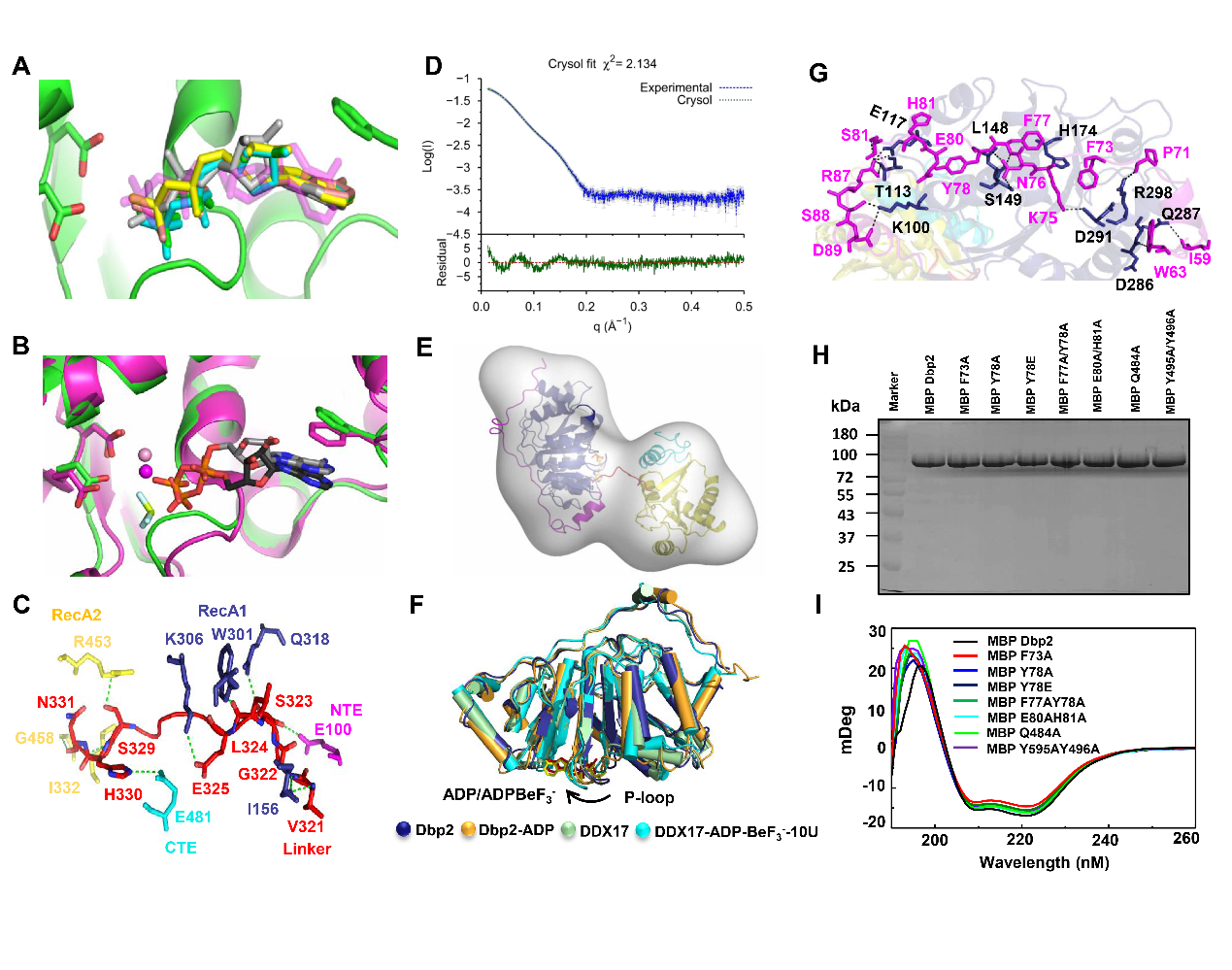


**Figure S4**. The crystal structure of Dbp2-apo maintains a rigid conformation through interactions among domains, related to Figures 2 and 3. **A**, variability of ADP position in the six chains of Dbp2-ADP. Orientation is conserved except for chain C. average B-factors for ADP in chain A to F are respectively (in Å^2^) 73.0, 110.5, 90.4, 102.5, 94.9, and 75.7. Chain A green, chain B cyan, chain C magenta, chain D yellow, chain E salmon, chain F grey. **B**, comparison of nucleotide binding site of Dbp2 (protein in green, ADP in light grey and Mg ion in pink) and DDX17 (PDB 6uv1) (protein in magenta, ADP in dark grey, Mg ion in magenta) shows a good conservation of the nucleotide-binding site. **C,** a detailed view of the interaction between the linker and other domains. Contact residues are shown as sticks. Hydrogen bonds are presented as green dashes. **D**, a fit curve of SAXS data of Dbp2^53-496^-ADP. **E**, crystal structure of Dbp2^53-496^-ADP was fitted into the ab initio envelope obtained from SAXS. **F**, superposition of the RecA1 domains of Dbp2-apo (deep blue), Dbp2-ADP (bright orange), DDX17-apo (pale green), DDX17/ADP·BeF_3_^-^-U10 (cyan), P loop adopts different conformations. ADP is shown as red sticks and ADP·BeF_3_^-^ is shown as yellow sticks. **G**, a detailed view of the interaction between NTE and RecA1 domain. Contact residues are shown as sticks. Hydrogen bonds are presented as black dashes. **H**, 10% SDS-PAGE analyses of Dbp2 and mutant full-length proteins. The wild-type and mutant protein contain a MBP tag. **I**, CD spectra analyses of Dbp2 and mutant full-length proteins. The wild-type and mutant protein contain a MBP tag.


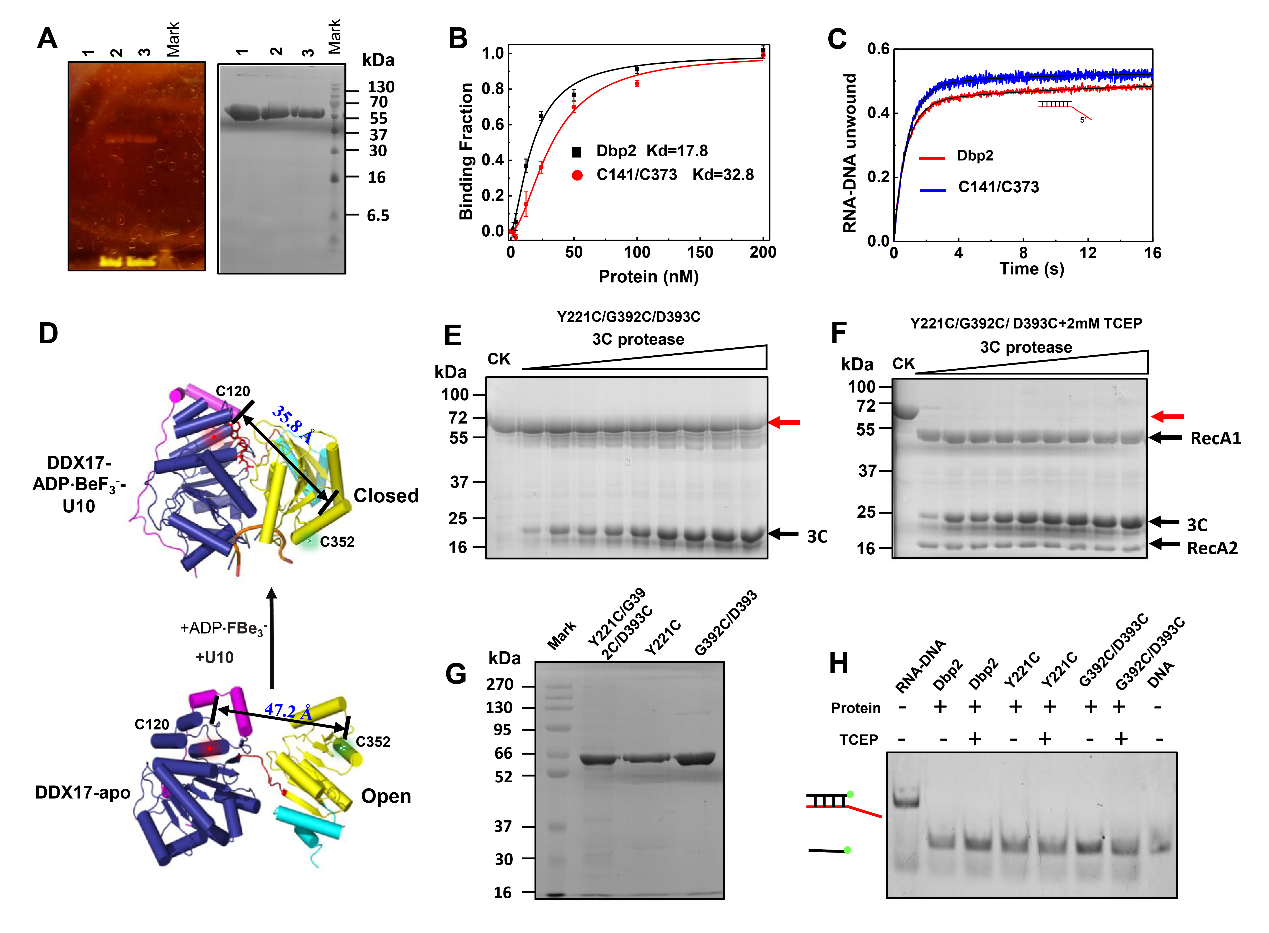


**Figure S5.** The binding and unwinding activities of mutants are related to Figure 4. **A**, gel analysis of C141/C373 with labeled Cy3 and Cy5, left is the fluorescence image, right is the white light image (1: protein before labeling; 2: the labeled protein; 3: purified labeled protein). C141/C373 contains a His_6_ tag at the C-terminus. **B**, the binding curves of WT Dbp2 and C141/C373 to MycG4 DNA. **C.** The unwinding activity of WT Dbp2 and C141/C373 to 5' overhang RNA-DNA hybrid duplex using stopped-flow assays. **D**, we measured the distances between C120 and C352 of DDX17 in the open and closed states. C120 and C352 in DDX17 correspond to C141 and C373 in Dbp2, respectively. Because of DDX17-ADP**^.^**BeF_3_^-^-U10 ternary complex structure is the closed state of the helicase core. **E**, testing the extent of disulfide bond formation of Y221C/G392C/D393C in the absence of TCEP by SDS-PAGE. The red arrow shows that Y221C/G392C/D393C was not digested by 3C protease. **F**, testing the extent of disulfide bond formation of Y221C/G392C/D393C in the presence of TCEP by SDS-PAGE. **G.** purity analyses of Dbp2 mutant proteins by SDS-PAGE. **H**, the unwinding activity of Dbp2 and mutants using 5' overhang RNA-DNA hybrid duplex.


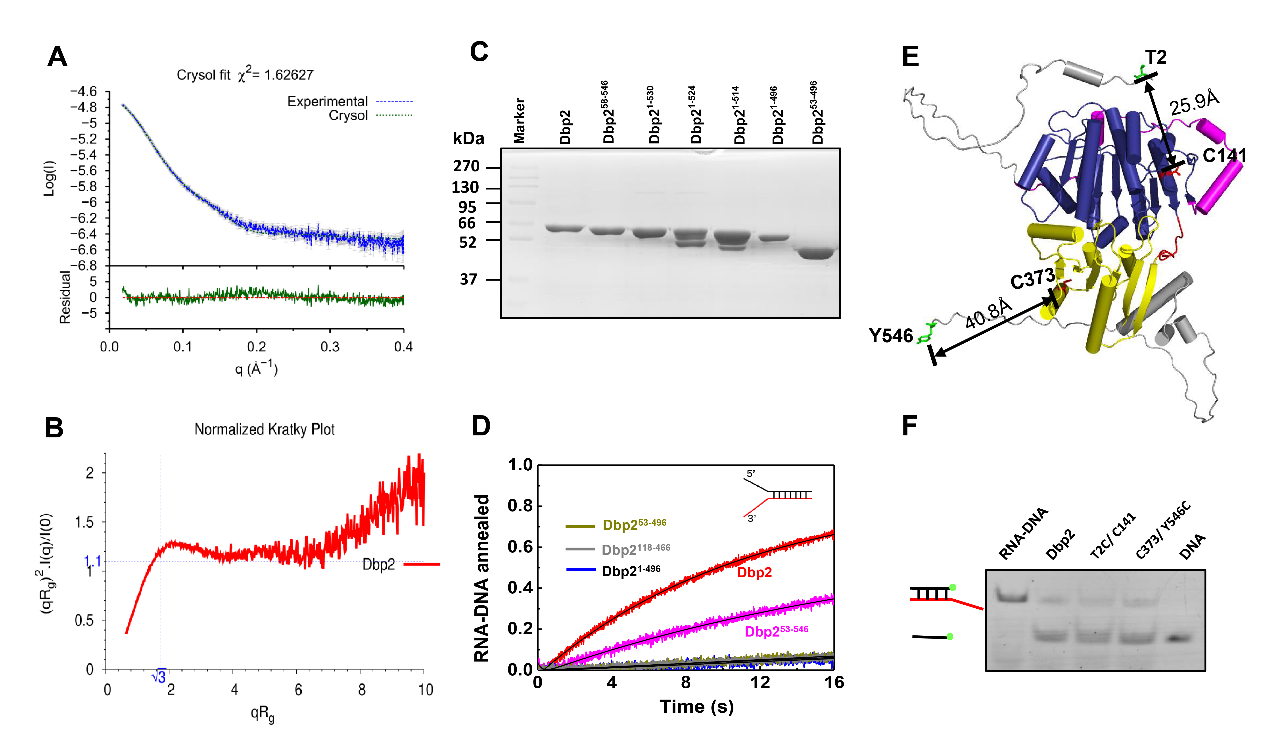


**Figure S6.** Measuring the activities of Dbp2 and several truncations related to Figures 5 and 6. **A**, a fit curve of SAXS data of full-length Dbp2. **B**, the Kratky plot shows full-length Dbp2 is a disorder in solution. **C**, purity analysis of truncated proteins by SDS-PAGE. **D**, the annealing activity of Dbp2 truncation proteins using fork RNA-DNA duplex in the stopped-flow kinetics assay. **E**, the AlphaFold predicts the distances of T2 and C141 and the distances of C373 and Y546. **F**, the unwinding activity of Dbp2 and mutants using 5' overhang RNA-DNA hybrid duplex.


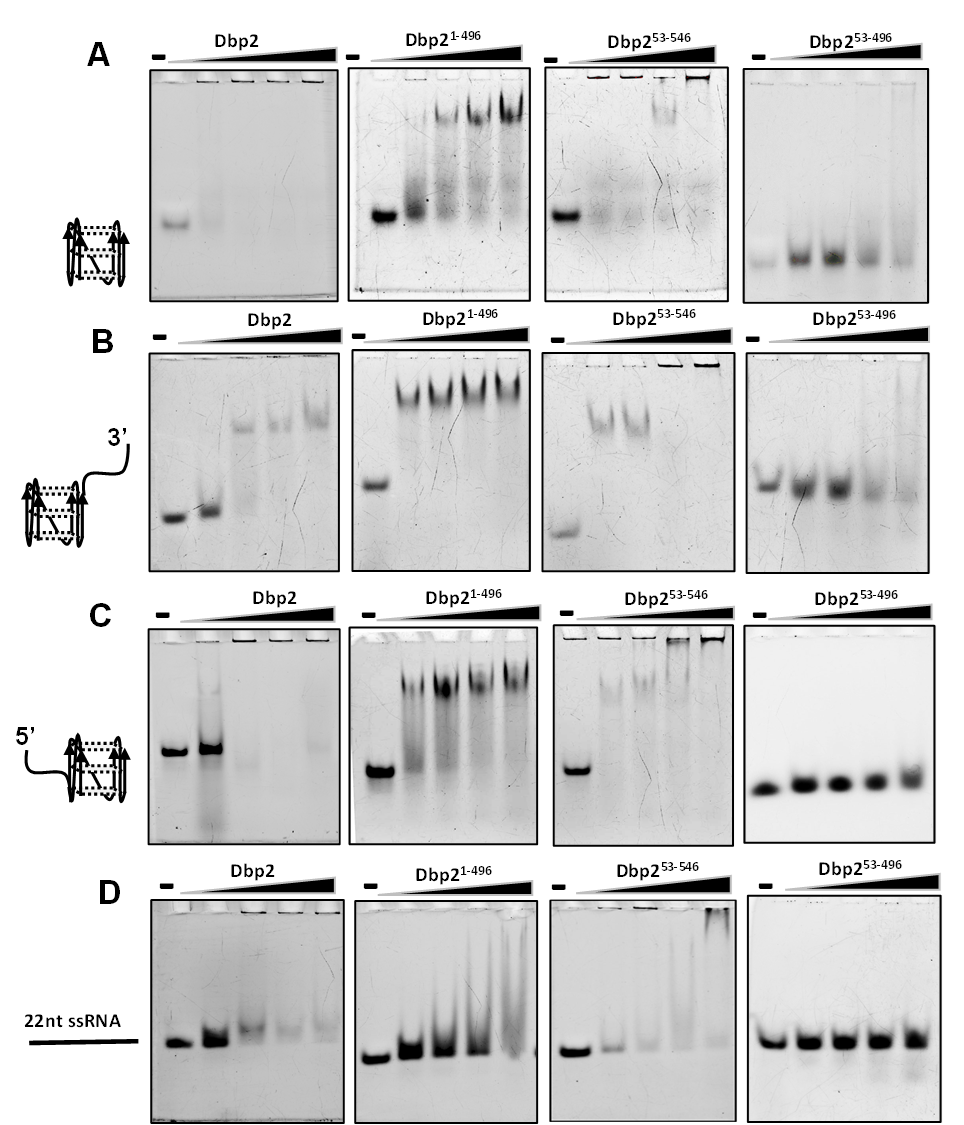


**Figure S7.** The binding activities of Dbp2 and truncated proteins are related to Figure 6. The binding affinities of Dbp2 truncated proteins measured to different substrates R-G4^Tel^ (A) 3'R-G4^Tel^ (B) 5'R-G4^Tel^ (C) R-S22 (D) by EMSAs. Protein concentrations of Dbp2 are (left to right): 0, 0.2, 0.4, 0.6, 0.8 μM; Protein concentrations of Dbp2^1-496^, Dbp2^53-546^, and Dbp2^53-496^ are (left to right): 0, 0.5, 1.0, 3.0, 6.0 μM;

**Table S1.** Binding, ATPase, unwinding and annealing characteristics of Dbp2.

|  | **Binding** | **ATPase** | **Unwinding parameter** | | **Annealing parameter** | |
| --- | --- | --- | --- | --- | --- | --- |
| **Substrate** | ***K*_d_(nM)** | **(μmol ADP/min/μM Dbp2)** | ***Rate* (s^-1^)** | ***Amplitude*** | ***Rate* (s^-1^)** | ***Amplitude*** |
| **R-G4^Tel^** | 12.2±0.49 |  |  |  |  |  |
| **R-S22** | 15.8±0.66 | 15.60±2.34 |  |  |  |  |
| **R/D-D16** | 40.56±4.65 | 18.39±0.42 |  |  |  |  |
| **R/D-D12** | 73.87±3.93 | 5.68±0.43 |  |  |  |  |
| **R-S12** | 72.14±5.09 | 1.65±0.51 |  |  |  |  |
| **D-S12** | 104.99±1.9 | 1.5±0.79 |  |  |  |  |
| **D-D12** | 178.3±6.2 | 1.04±0.51 |  |  |  |  |
| **D-Y** |  |  | ND | ND | 0.13±0.01 | 0.67±0.01 |
| **R-5′Oh** |  |  | 0.06±0.01 | 0.20±0.01 | 0.14±0.01 | 0.68±0.01 |
| **R-3′Oh** |  |  | 0.06±0.01 | 0.22±0.01 | 0.14±0.01 | 0.61±0.01 |
| **R-D12** |  |  | 0.06±0.01 | 0.14±0.01 | 0.41±0.02 | 0.89±0.02 |
| **R-Y** |  |  | 0.02±0.01 | 0.14±0.01 | 0.18±0.01 | 0.77±0.01 |
| **R/D-D12** |  |  | 0.28±0.04 | 0.63±0.02 | 0.26±0.02 | 0.89±0.01 |
| **R/D-5′Oh** |  |  | 0.54±0.06 | 0.50±0.06 | 0.17±0.05 | 0.60±0.15 |
| **R/D-3′Oh** |  |  | 0.32±0.01 | 0.64±0.01 | 0.04±0.02 | 0.40±0.01 |
| **R/D-Y** |  |  | 0.53±0.01 | 0.91±0.01 | 0.16±0.03 | 0.66±0.02 |

The assays were completed to determine mean values and standard deviations (n ≥3). ND, no detection.

**Table S2.** Crystallographic data collection and refinement statistics.

| **Data collection** | Dbp2^53-496^ | Dbp2^53-496^-ADP | **Refinement** | Dbp2^53-496^ | Dbp2^53-496^-ADP |
| --- | --- | --- | --- | --- | --- |
| Space group | C 1 2 1 | P 2_1_ 2_1_ 2_1_ | R-work /R-free | 0.224 (0.252) | 0.222 (0.271) |
| Unit cell | 204.10 115.12 81.41 90 101.92 90 | 145.94 168.84 168.87 90 90 90 | Number of non-hydrogen atoms | 9085 | 21294 |
| Wavelength | 0.9778 | 0.9793 | Macromolecules | 9085 | 21089 |
| Resolution （Å） | 38.85 - 3.22 (3.33 - 3.22) | 119.4 - 3.05 (3.16 - 3.05) | ligands | 0 | 205 |
| Total reflections | 97964 (10196) | 1037563 (103433) | RMS (bonds) (Å) | 0.005 | 0.005 |
| Unique reflections | 28601 (2971) | 77028 (7932) | RMS (angles) (°) | 1.04 | 1.00 |
| Multiplicity | 3.4 (3.4) | 13.5 (13.0) | Ramachandran Favored (%) | 99.30 | 98.91 |
| Completeness (%) | 94.47 (98.50) | 96.19 (100.00) | Ramachandran allowed (%) | 0.61 | 0.98 |
| Mean I/sigma(I) | 15.17 (2.63) | 17.34 (2.45) | Ramachandran outliers (%) | 0.09 | 0.11 |
| Wilson B-factor (Å^2^) | 101.06 | 98.05 | Average  B-factor (Å^2^) | 134.70 | 109.63 |
| R-merge | 0.06332 (0.5775) | 0.1607 (1.094) | macromolecules | 134.70 | 109.80 |
| CC1/2 | 0.998 (0.812) | 0.996 (0.734) | ligands |  | 91.22 |
| ^a^Statistics for the highest-resolution shell are shown in parentheses. | | | | | |

**Table S3.** SAXS data collection and processing statistics of Dbp2^53-496^ and Dbp2^53-496^-ADP.

| **Dbp2**^53-496^ **Dbp2^53-496^-ADP** | | | **Dbp2**^53-496^ **Dbp2^53-496^-ADP** | | | |  |
| --- | --- | --- | --- | --- | --- | --- | --- |
| **Data-collection parameters** | |  | **Molecular-mass determination** | | | |  |
| Instrument | BL19U2 | SWING | | Partial specific volume (cm^3^.g^-1^) | 0.74 | 0.74 | |
| Beam geometry (mm) | 0.33x0.05 | 0.4×0.1 | | Contrast (Δρ×10^10^ cm^-2^) | 2.82 | 2.82 | |
| Wavelength (Å) | 1.03 | 1.03 | | Molecular mass Mr [from I (0)] | 67783 | 45000 | |
| q range (Å^-1^) | 0.007-0.4 | 0.007-0.5 | | Calculated monomeric Mr from sequence | 61455 | 49000 | |
| Detector | Pilatus 1M | Pilatus 1M | | **Data processing** |  |  | |
| Data collection mode | batch | HPLC | | Primary data reduction | BioXTAS RAW | FOXTROT | |
| Exposure time (s) / nb frames | 1.5 / 20 | 1/540 | | Data processing | PRIMUS | PRIMUS | |
| Concentration (injected) (mg.ml^-1^) | 5 | 10 | | Ab initio analysis | DAMMIF | DAMMIF | |
| Temperature (K) | 288 | 288 | | Number of models | 50 | 50 | |
| **Structural parameters** |  |  | | Model χ^2^ | 1.254 ± 0.002 | 1.27 ± 0.03 | |
| Guinier quality: |  |  | | Validation and averaging | DAMAVER | DAMAVER | |
| data points | 6-45 | 69 | | NSD | 1.26 ± 0.14 | 0.91 ± 0.22 | |
| qRg | 0.47-1.23 | 1.29 | | Estimated resolution (Å) | 39 ± 3 | 33 | |
| correlation coefficient | 0.9923 | 0.9994 | | Rigid-body modelling | EOM | DADIMODO | |
| I (0) (cm^-1^) [from Guinier] | 6.45e-08 ± 0.2e-08 | 0.0614± 0.0001 | | Computation of model intensities | CRYSOL | CRYSOL | |
| Rg (Å) [from Guinier] | 37.6 ± 1.8 | 29.24 ± 0.02 | | Model χ^2^ | 1.62 | 2.13 | |
| Rg (Å) [from P(r)] | 37.5 | 29.26 | |  |  |  | |
| Dmax (Å) | 132.1 | 101.2 | |  |  |  | |
| Porod estimate (Å^3^) | 108453 | 71976 | |  |  |  | |

**Table S4.** Interaction residues of NTE and distances of hydrogen bonds in the structures of Dbp2-apo, Dbp2-ADP, DDX17-apo, and DDX17-ADP·BeF_3_^-^-U10.

| **Structure** | **NTE** | **RecA1** | **RecA2** | **Linker** | **Distance** |
| --- | --- | --- | --- | --- | --- |
|  | E466 |  | T369 |  | 2.6 Å |
|  | T465 |  | V339 |  | 2.9 Å |
|  | N468 |  | M437 |  | 3.4 Å |
| **Dbp2-apo** | S477 |  | T159 |  | 3.5 Å |
|  | E481 |  |  | H330 | 3.4 Å |
|  | Q484 |  | I332、Q334 |  | 2.8 Å、3.5 Å |
|  | R495 | E131 |  |  | 3.0 Å |
|  | R496 | V128、T137 |  |  | 3.1 Å、3.4 Å |
| **Dbp2-ADP** | F464 |  | E337、M437 |  | 2.9Å、2.6 Å |
|  | N468 |  | V339 |  | 3.2 Å |
|  | Q484 |  |  | I332 | 3.2 Å |
| **DDX17-apo** | Q450 |  | P417 |  | 3.4 Å、2.8 Å |
|  | E453 |  | S420 |  | 3.5 Å |
|  | N462 | K282 |  |  | 3.3 Å |
|  | Q463 |  | Q313 | I311 | 3.1 Å、3.3 Å |
|  | N466 |  | V315 |  | 2.3 Å |
|  | D474 |  |  |  | 1.9 Å |
| **DDX17-ADP·BeF_3_^-^**  **-U10** | F443 |  | D316 、C318 |  | 2.6 Å 2.8 Å |
|  | T444 |  | E320 |  | 3.3 Å |
|  | Q450 |  | P417 |  | 2.8 Å |
|  | Q463 |  | Q313 |  | 3.2 Å |
|  | E460 |  |  | N302、H309 | 3.5 Å、3.4 Å |
|  | N466 |  | V315 |  | 2.6 Å |

**Table S5.** Unwinding parameters of Dbp2 and mutants in the stopped-flow kinetics assay using RNA-DNA duplex with 5' overhang.

| **P**  **P** | **Binding** | **Unwinding parameter** | |
| --- | --- | --- | --- |
|  | ***K*_d_ (nM)** | ***Rate* (s^-1^)** | ***Amplitude*** |
| **Dbp2** | 72.3±3.8 | 0.54±0.06 | 0.50±0.06 |
| **Dbp2^F73A^** | ND | 0.04±0.01 | 0.17±0.12 |
| **Dbp2^F77AY78A^** | ND | ND | ND |
| **Dbp2^Y78A^** | ND | ND | ND |
| **Dbp2^Y78E^** | 280.7±11.6 | ND | ND |
| **Dbp2^E80AH81A^** | 116.4±18.6 | 0.23±0.01 | 0.43±0.04 |

The three independent assays were completed to determine mean values and standard deviations. P: parameters; S, substrate; P, protein; ND, no detection.

**Table S6.** Binding, unwinding and annealing parameters of Dbp2 and truncated proteins in the stopped-flow kinetics assay using a forked RNA-DNA duplex.

| **P P** | **Binding *K*_d_ (nM)** | | **ATPase** | **Unwinding parameter** | | **Annealing parameter** | |
| --- | --- | --- | --- | --- | --- | --- | --- |
|  | **R-S22** | **R-G4^Tel^** | **(μmol ADP/min/μM Protein)** | ***Rate* (s^-1^)** | ***Amplitude*** | ***Rate* (s^-1^)** | ***Amplitude*** |
| **Dbp2** | 15.8±0.66 | 12.2±0.49 | 18.39±0.42 | 0.47±0.02 | 0.99±0.15 | 0.15±0.04 | 0.77±0.02 |
| **Dbp2^1-496^** | 244.62±14.07 | 154.79±13.91 | 4.85±0.03 | 0.12±0.01 | 0.67±0.01 | ND | ND |
| **Dbp2^53-546^** | 64.8±19.01 | 18.8±0.56 | 11.97±0.45 | 0.35±0.01 | 0.98±0.02 | 0.04±0.02 | 0.34±0.02 |
| **Dbp2^53-496^** | ND | ND | 1.41±0.14 | 0.005±0.01 | 0.09±0.02 | ND | ND |
| **Dbp2^118-466^** | ND | ND | ND | ND | ND | ND | ND |
| **Dbp2^1-514^** | 214.13±18.40 | 63.46±8.45 |  |  |  |  |  |
| **Dbp2^1-524^** | 38.76±4.35 | 27.85±2.66 |  |  |  |  |  |
| **Dbp2^1-530^** | 33.69±6.63 | 19.16±2.17 |  |  |  |  |  |

The assays were completed to determine mean values and standard deviations (n ≥3). P: parameters; P, protein; ND, no detection.

**Supplementary Table7.** DNA and RNA sequence used in different assays.

| **Assay** | **Name** | **Sequence (5'-3')** |
| --- | --- | --- |
| **Fluorescence anisotropy assay and EMSA assay** | **R-S12** | CUCUGCUCGACG-**Fam^a^** |
|  | **R-S22** | UUUUUUUUUUCUCUGCUCGACG-**Fam** |
|  | **D-S12** | ACGGATGTCTAA-**Fam** |
|  | **R/D-D16** | GAUCGUGACAUCCUUA |
|  |  | TAAGGATGTCACGATC -**Fam** |
|  | **R/D-D12** | CGUCGAGCAGAG |
|  |  | CTCTGCTCGACG-**Fam** |
|  | **D-D12** | CGTCGAGCAGAG |
|  |  | CTCTGCTCGACG-**Fam** |
|  | **R-G4^Tel^** | GGGUUAGGGUUAGGGUUAGGG-**Fam** |
|  | **3'R-G4^Tel^** | UUUUUUUUUUUUGGGUUAGGGUUAGGGUUAGGG-**Fam** |
|  | **5'R-G4^Tel^** | GGGUUAGGGUUAGGGUUAGGGUUUUUUUUUUUU-**Fam** |
| **Unwinding and annealing assay** | **R-D12** | CUCUGCUCGACG-**Fam**  **HF^b^**-CGUCGAGCAGAG |
|  | **R-3′Oh** | **HF**-CGUCGAGCAGAGUUUUUUUUUU  CUCUGCUCGACG-**Fam** |
|  | **R-5′Oh** | **HF**-CGUCGAGCAGAG  UUUUUUUUUUCUCUGCUCGACG-**Fam** |
|  | **R-Y** | UUUUUUUUUUCUCUGCUCGACG-**Fam**  **HF**-CGUCGAGCAGAGUUUUUUUUUU |
|  | **R/D-D12** | **HF-**CGUCGAGCAGAG  CTCTGCTCGACG-**Fam** |
|  | **R/D-3′Oh** | **HF**-CGUCGAGCAGAGUUUUUUUUUU  CTCTGCTCGACG-**Fam** |
|  | **R/D-5′Oh** | **HF-**CGTCGAGCAGAG  UUUUUUUUUUCUCUGCUCGACG-**Fam** |
|  | **R/D-Y** | UUUUUUUUUUCUCUGCUCGACG**-Fam**  **HF-**CGTCGAGCAGAGTTTTTTTTTT |
|  | **D-Y** | **HF-**CGTCGAGCAGAGTTTTTTTTTT CACTGGCCGTCTTACGGTCGCTCTGCTCGACG**-Fam** |
| **smFRET assay** | **R-G4^Tel*^** | GGGUUAGGGUUAGGGUUAGGG |
|  | **3'R-G4^Tel*^** | UUUUUUUUUUUUGGGUUAGGGUUAGGGUUAGGG |
|  | **R-S22*** | UUUUUUUUUUCUCUGCUCGACG |
|  | **D-D12*** | CGTCGAGCAGAG  CTCTGCTCGACG |

^a^Fam, fluorescein.

^b^HF, hexachlorofluorescein.

*, the substrate used for smFRET experiment.
